# Robust multiclass classification of crop leaf diseases using hybrid deep learning and Grad-CAM interpretability

**DOI:** 10.1038/s41598-025-14847-7

**Published:** 2025-08-15

**Authors:** Sankar Murugesan, Jayaprakash Chinnadurai, Saravanan Srinivasan, Sandeep Kumar Mathivanan, Radha Raman Chandan, Usha Moorthy

**Affiliations:** 1 Department of Electronics and Communication Engineering, Vel Tech Rangarajan Dr. Sagunthala R&D Institute of Science and Technology, Avadi, Chennai, Tamilnadu India; 2 Department of Computer Science and Engineering, SRM Institute of Science and Technology, Ramapuram, Chennai, 600089 Tamilnadu India; 3 School of Computer Science and Engineering, Galgotias University, Greater Noida, 203201 India; 4 Department of Computer Science, School of Management Sciences (SMS), Varanasi, 221011 UP India; 5 School of Computer Engineering, Manipal Institute of Technology, Bengaluru,, Manipal Academy of Higher Education, Manipal, India

**Keywords:** Plant leaf disease, Classification, ConvNet, Deep learning, Vision transformer, Hybrid ConvNet-ViT, Plant sciences, Plant cell biology

## Abstract

The key objective of this study is to propose an effective and accurate deep learning (DL) framework to detect and classify diseases in banana, cherry, and tomato leaves. The performance of multiple pre-trained models is compared against a newly presented model.The experiments used a publicly released dataset of healthy and unhealthy leaves from banana, cherry, and tomato plants. This dataset was uniformly split into training, validation, and test sets to obtain consistent and unbiased model evaluations. The data pre-processing also involved pre-processing steps suitable for DL architectures to keep the input the same among all the models.We use several state-of-the-art pre-trained ConvNets models for the baselines, such as EfficientNetV2, ConvNeXt, Swin Transformer, and Vi-Transformer (ViT), to have an outlook on the performance. A new ConvNet-ViT hybrid model combines the ConvNet and ViT layers for local feature extraction and maintaining the global context. The classifier’s performance was reinforced by a 5-fold cross-validation mechanism to avoid overfitting.The proposed Hybrid ConvNet-ViT model outperformed all the compared models evaluated, achieving a testing classification accuracy of 99.29%, which outperforms all the pre-trained models. This finding shows that combining ConvNets’ local feature learning with the capability of global representation of the ViT is effective.The result shows that the Hybrid ConvNet-ViT model is an effective and accurate solution in detecting and classifying plant leaf diseases. Its outstanding performance of the state-of-the-art pre-trained top models positions itself as a solid model for practical agricultural use. Fusing the ConvNet and transformer frameworks jointly is beneficial for improving classification performance in image-based disease detection work.

## Introduction

Banana, cherry, and tomato are some of the most important horticultural crops grown worldwide due to their nutritional, economic, and commercial value^1^. These crops are vital for food security and have served as an essential source of income for millions of farmers in several agro-ecological zones^2^. Bananas are necessary in tropical and subtropical areas of the world, where they are eaten raw and in various processed forms. Cherries are an antioxidant-rich fruit commonly consumed fresh and processed into jams, juices, and pastries in temperate regions^3^. Tomatoes are among the most widely consumed and versatile fruits in the world, and it’s no wonder because they’re not only delicious and nutritious, but are an excellent source of vitamins A, C, and K, potassium, and lycopene. As they are widely cultivated and are of high commercial importance, their health and productivity, compromised by diseases, affect the food systems and economies in the rural areas and beyond^4^. One of the most significant threats to the growth of banana, cherry, and tomato plants is foliar disease -- diseases that infect the leaves of the plants^5^. Foliar diseases are typically more destructive, as they reduce photosynthetic capacity, weaken plant health, and are often a door for secondary infections^6^. In banana plants, Black Sigatoka is a significant disease, especially in hot environments, and can decrease the leaf surface area and shoot cycle, influencing yields directly^7^. Similarly, cherry plants are frequently attacked by Cherry Leaf Spot, a disease that causes severe defoliation and significantly lowers fruit quality and quantity. Tomato plants are highly vulnerable to a broad spectrum of leaf diseases, such as Early and Late Blight, capable of decimating entire crops within days under favourable weather conditions^8^. These diseases are generally exacerbated by the high humidity, unsuitable crop rotation practices, and global climate change, which influence the occurrence and virulence of pathogens. These leaf diseases are also transmitted through wind, water splashes, insects, tools, or soil with pathogens^9^. When introduced, they can infest fields quickly, especially for mass cultivation, making routine visual checking of infected plants very costly. Recognition of leaf diseases by traditional ways depends on experts, is time-consuming, subjective, and inconsistent^10^.

The shared diagnostic symptoms among different diseases, between various diseases, and even with non-disease stresses, such as nutrient deficiency or pesticide damage, make recognizing the diseases even more challenging. In addition, rural or underdeveloped areas may not have easy access to trained agronomists and laboratories for correct plant disease diagnosis^11^. Artificial intelligence (AI), computer vision, and deep learning have recently arisen as an excellent alternative for plant disease detection and classification in these settings. These methods, especially the digital imagery of leaves, can automatically identify complex features and characteristics distinct in healthy and infected leaves with certain diseases^12^. ConvNets have shown significant promise in capturing local features such as edges, spots, and textures. At the same time, Transformer-based models like ViTs excel at capturing long-range dependencies and contextual relationships within images. The fusion of these two architectures into hybrid models offers an exciting new direction, combining the strengths of both local and global feature representations^13^. Early and precise leaf disease detection using AI-based models helps minimize crop damage. It allows fungicides or bio-control agents to be sprayed precisely at the right time, facilitating compliance with environmental laws and reducing production costs. These can be mounted on mobile phones or drones so farmers can diagnose diseases and pests in real-time^14^. With increasing needs for sustainable and resilient agriculture, deep learning based automated plant disease diagnostics has strong potential in revolutionizing conventional crop monitoring and guaranteeing agricultural sustainability in the long run. The health of leaves is an essential aspect of a plant’s fitness. Visiting leaves’ color, texture, and shape changes is necessary for detecting diseases and deciding on irrigation, fertilization, and pesticide application^15^. Early detection reduces yield losses and leads to more sustainable farming by limiting broad-spectrum chemical applications. The rapid decision-based farmer’s agriculture and limited-resource-based smallholder’s agriculture need practical, timely plant health monitoring systems in commercial and smallholder-based agricultures. As agriculture further modernizes and embraces data and precision farming, understanding the character and symptoms of foliar diseases in essential crops such as banana, cherry, and tomato is still one of the basic principles of good plant health management^16^.

The proposed research aims to develop an AI solution that automatically detects and classifies plant leaf disease from field images using state-of-the-art deep learning methods, and can support early detection and agricultural decision-making. Leaf diseases in banana, cherry, and tomato represent a significant threat to the health of the plants as they can decrease the photosynthetic efficiency and therefore decrease yields. The conventional means of identifying diseases visually, using the human eye is time-consuming, open to human error, and unavailable to many growers in remote or less-resourced locations. This study addresses these challenges by developing an image-based detection system capable of accurately identifying visual symptoms of disease from leaf images. The models integrate ConvNet layers and attention-based mechanisms, such as those used in vision transformer architectures, to capture local and global features. This combination enhances the system’s ability to recognize diseases that vary in appearance depending on their stage of development or environmental conditions. The objective is to create a unified model architecture that can effectively interpret leaf imagery, regardless of crop type or disease variation.

The organization of this study is structured as follows. Chapter 2 discusses recent state-of-the-art crop leaf disease classification models, highlighting their advantages and limitations. Chapter 3 presents the materials and methods; the materials section provides a detailed overview of the dataset, while the methods section explains the architectures of both the pre-trained and proposed models. Chapter 4 outlines the experimental results, including classification accuracy for both model types. Chapter 5 focuses on the ablation study, analyzing the impact of various components in the proposed approach. Chapter 6 provides a comparative analysis of performance metrics between the proposed model and other state-of-the-art models. Finally, Chap. 7 concludes the study by summarizing the efficacy of pre-trained and proposed models in disease classification and discusses potential directions for future research.

## Related work

Nixon Jiménez et al.^17^ study used the CRISP-DM approach to process 900 images of banana leaves. Three pre-trained models (EfficientNetB0, ResNet50, and VGG19) were re-trained using this dataset. Data augmentation was used to improve the model’s performance; the new dataset size was 9,000 pictures. EfficientNetB0 had the highest classification accuracy of 88.33%, 88.90%, and 87.22% in classifying diseases, indicating that it was the most powerful in classifying banana leaf diseases.

Joshva Devadas Thiagarajan et al.^18^ study proposes two strategies for improving disease prediction and detection on banana leaves. Current models are prone to rotation and scale invariance; thus, ConvNets have been explored with the aim of better prediction. As an alternative, the paper presents two hybrids, one of ANN with sift and the other of hog with LBP. The first model leverages activation functions to process SIFT extracted features, whereas the second model concatenates HOG and LBP features for analysis.

Christian A. Elinisa et al.^19^ research has investigated applying a U-Net-based semantic-segmentation model for early detection and segmentation of Fusarium Wilt and Black Sigatoka in Banana. They trained the model with a dataset of 18,240 labelled images of banana leaves and stalks showing signs of these diseases. The pictures were taken on farms with mobile phone cameras, accompanied by agricultural professionals. The U-Net model achieved high performance with a Dice Coefficient of 96.45% and an intersection over Union (IoU) score of 93.23%.

Christian A. Elinisa et al.^20^ study indicates the capacity of deep learning in the early diagnosis of banana diseases. Such a system could adeptly differentiate between healthy and infected banana leaves and stalks and be able to recognize non-banana plant images with more than 90% confidence within 5 s per image. It also provided evidence-based suggestions for the control of diseases.

Emrah Dönmez1 et al.^21^ systems aid in monitoring each phase of agriculture - from planting, growth, and harvesting - to make informed decisions for profitable crops. One essential task of such systems is to detect and monitor plant diseases. In their study, cherry plant diseases are detected by ConvNets, with DarkNet-19 being the model used. Machine learning-derived features are extracted and used to identify irritations. These extracted features are then classified with various linear and non-linear classifiers to handle a multi-class classification problem between healthy, mildly diseased, and severely diseased plants. The detection accuracy results show a detection rate of 88.1%, which is better.

Manjunatha Shettigere Krishna et al.^22^ study aims to create models that can precisely detect plant diseases in different environmental conditions, rectifying existing practices. A merged dataset was created by combining the PlantDoc dataset with plant images from various online platforms. A further refinement was done with the state-of-the-art ConvNet architectures - EfficientNet-B0, EfficientNet-B3, ResNet50, and DenseNet201 for plant leaf disease classification. One significant contribution of their work is utilizing powerful data augmentation methods, such as the injection of Gaussian noise, to facilitate model generalization. The models performed differently depending on the datasets. When trained and tested only on PlantDoc, EfficientNet-B3 achieved 73.31% accuracy. The model demonstrated 76.77% accuracy on the cross-data-set testing. The best accuracy of 80.19% was achieved in the combined dataset, explaining that the model had good generalization for diverse conditions.

Yong Wang et al.^23^ also applies attention mechanisms, multi-scale feature fusion to a method for tomato leaf disease detection. A backbone feature extraction network is adopted, incorporating the Convolutional Block Attention Module (CBAM) to enhance the feature extraction from the lesion and decrease the noise from outside. Then, the shallow feature maps are put into the re-parameterized generalized feature pyramid network (RepGFPN), and a new multi-scale fusion module is named BiRepGFPN. The BiRepGFPN substitutes the conventional PAFP-Feature pyramid network (PAFPN) in the YOLOv6 to further incorporate deep semantic and surface spatial features. Experimental results on the PlantDoc demonstrated that the proposed model could effectively surpass conventional approaches by 7.7%, 11.8%, 3.4%, 5.7%, 4.3%, and 2.6% in mAP over YOLOX, YOLOv5, YOLOv6, YOLOv6-s, YOLOv7, and YOLOv8, respectively. On the tomato leaf disease dataset, the model’s accuracy was 92.9%, the recall was 95.2%, the F1 score was 94.0%, and the mAP was 93.8%. In comparison, the improvements over the baseline model were 2.3%, 4.0%, 3.1%, and 2.7%, respectively.

Vengaiah Cheemaladinne et al.^24^ study integrates superior deep learning and VARMAx models, which can efficiently and accurately detect tomato leaf diseases. This proactive strategy provides a powerful possibility for superior classification and optimal post-processing, which solves the major issues of modern agriculture. The key component of the proposed solution is the VARMAx-ConvNet-GAN Integration architecture. ConvNet is in charge of feature extraction and disease classification in this model. Meanwhile, additional synthetic images are generated by generative adversarial networks (GANs) to enrich the training set and enhance generalization. The joint model helps improve the precision and robustness in the identification of diseases, and has the potential to provide improved decision support to farmers and experts in agriculture. Precision, Accuracy, Recall, AUC, and speed of the detection of tomato diseases. The results indicate that compared with traditional methods, the method used in this paper has higher precision, accuracy, recall, AUC, and faster detection rates of tomato diseases.

Rashid Khan et al.^25^ utilized feature extraction methods like GLCM and SIFT along with Support Vector Machine (SVM) designed for tomato leaf disease detection and classification. Initially employed a 2,700-image dataset containing no less than 300 samples for any disease category. The algorithm was highly accurate and reliable, improving the disease’s detection and classification. The framework is promising for tomato cultivation, but future work could be done concentrating on adopting advanced deep learning models.

Xuewei Wang et al.^26^ study presents a novel detection approach, TomatoDet, to detect plant diseases in the complex environment. The method employs a feature extraction module, Meta-ACON, and an Improved Bidirectional Weighted Feature Pyramid Network to achieve higher precision and lower error. Our method achieved 92.3% mean AP on a hand-designed dataset, an 8.7% AP improvement compared to our baseline model.

Mehdhar S.A.M et al.^27^ present a hybrid approach of plant disease classification which integrates transfer learning with Gravitational Search Algorithm (GSA). MobileNetV2 and ResNet50V2 extract features, which are then fused and optimized. Utilize GSA for fused feature. The features are discriminated using MLR on 18 classes (healthy and disease leaves). Comparative analysis with Genetic Algorithm (GA) and K-Nearest Neighbors (KNN) has different performances compared to other comparative models, where the GSA-MLR model has higher accuracy, and the average precision for MLR and KNN is 99.2% and 98.6% respectively.

Mehdhar S. A. M et al.^28^ demonstrate a plant disease classification model with Dilated Spatial Pyramid Pooling for a multi-scale image representation. Learned from diverse, augmented dataset of corn, coffee and other leaves The model generalizes well. It surpasses other approaches reaching a 99.34% precision, 99.25% recall, 99.29% F1-score and 99.35% accuracy.

Mehdhar S. A et al.^29^ present MSCPNet, a compact yet efficient model consisting of a compact MobileNetV2, followed by a Multi-Scale Convolutional PoolFormer block. The simplified backbone maintains the essential features, and multi-scale branches together with the PoolFormer module effectively learn both fine and coarse patterns. Targeting real-time maize disease detection, MSCPNet yields state-of-the-art results with 97.44% accuracy, 96.76% precision, 97.37% recall, 97.04% F1-score and MCC of 0.9653, with only 998 K parameters and 315 M FLOP.

**Table 1:** Comparative summary of existing methods.

Ref. no	Method used	Objective	Limitation
^17^	R-LSTM, C-LSTM	Time-series-based drowsiness detection	Ineffective on static images
^18^	ViT-G	Vision Transformer for eye closure	High computational cost
^19^	2D CNN + Eye aspect ratio	Image-based drowsiness detection	Limited feature extraction
^20^	Pre-trained DenseNet121	Binary fatigue classification	Lower accuracy on real-world images
^21^	Residual Channel-Attention Network	Channel-wise attention for fatigue	Overfitting due to small dataset
^22^	Fuzzy Inference System	Rule-based fatigue level estimation	Low generalization to diverse inputs
^23^	LSTM + CNN	Spatio-temporal fatigue modeling	High latency during inference
^24^	Attention CNN	Attention-enhanced eye closure detection	Performance drop under occlusion
^25^	YOLOv3 + Face Landmarks	Object detection + eye feature extraction	Lack of class-wise analysis
^26^	SVM + PERCLOS	Feature-based fatigue classification	Hard-coded feature thresholds
^27^	Eye Blink Detection CNN	Blink-based alertness detection	Cannot handle non-frontal faces
^28^	Hybrid CNN-GRU	Hybrid temporal classification	Training instability
^29^	MobileNetV2	Lightweight CNN for fatigue	Reduced accuracy on small datasets

As shown in Table 1, many existing drowsiness detection methods face significant limitations such as overfitting on small datasets, dependence on handcrafted features, or difficulty in capturing both spatial and temporal dependencies. CNN-only methods often lack a comprehensive understanding of the overall context, while RNN-based or transformer-heavy models tend to require high computational resources or face training stability issues. Some lightweight architectures also sacrifice accuracy under diverse real-world conditions. In contrast, the proposed hybrid ConvNet–ViT model effectively overcomes these challenges by combining local feature extraction with global context modelling. This balanced approach enables the model to accurately distinguish subtle differences between classes while maintaining computational efficiency. Additionally, the use of attention mechanisms, layer freezing strategies, and optimized training settings further improves generalization, making the proposed model suitable for multi-class drowsiness detection in real-world applications.

## Materials and methods

In this research, plant disease detection and identification are performed based on banana, cherry, and tomato leaf images. Various pre-trained convolutional and transformer models have been evaluated to compare the performance of the methods, which include: EfficientNetV2, ConvNeXt, Swin Transformer, and ViT. In addition to these existing architectures, we benchmarked a proposed Hybrid ConvNet-ViT model that combines ConvNets and vision transformer layers. The objective is to evaluate the performance of these models in terms of their ability to detect a range of leaf disease states. The subsequent sections provide a detailed description of the model architectures, implementation framework, evaluation, and performance analysis.

**Fig. 1:**
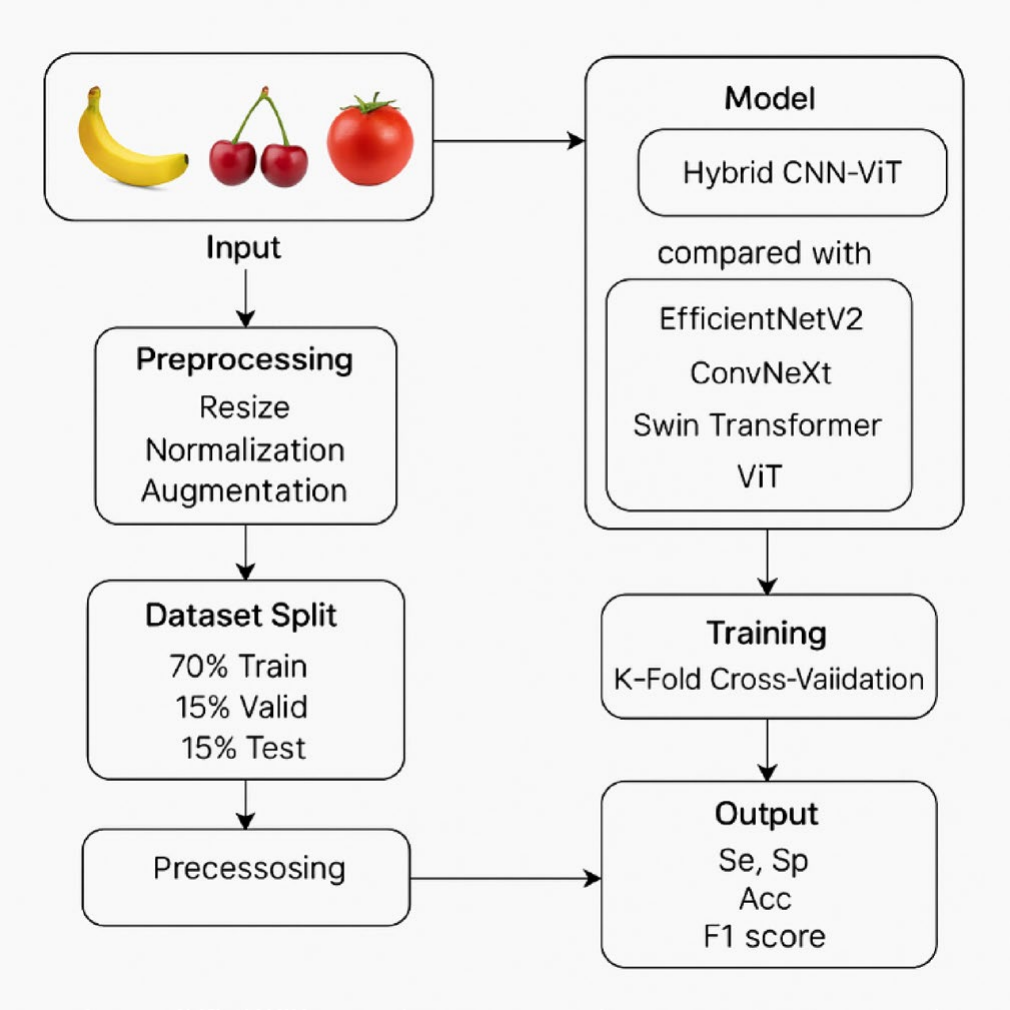
Overall architecture of proposed study.

### Materials

**Fig. 2:**
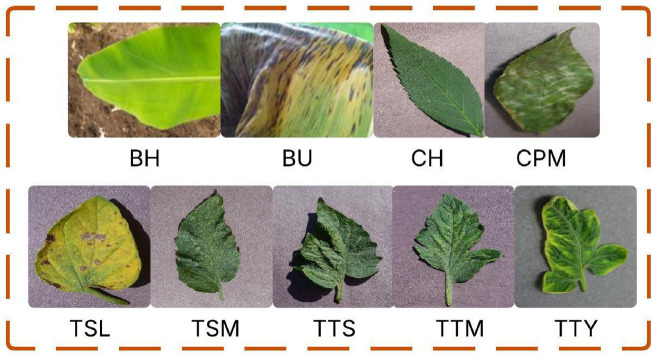
Sample dataset images of all diseases.

A publicly available dataset has been utilized in this study to evaluate the effectiveness of both pre-trained and proposed deep learning models for plant leaf disease detection and classification^30^. The dataset comprises images of banana, cherry, and tomato plant leaves, encompassing a variety of healthy and diseased conditions. To ensure the systematic comparison and fair performance assessment, the data set is tripartite into three parts, which are used for training (70%), validation (15%), and test (15%) in Table 2. This split enables iterative model optimization while in development and a proper, fair, and objective evaluation on unseen data. The dataset in this study consists of labeled images representing banana, cherry, and tomato leaves, and it is associated with nine classes, among which eight classes are for eight diseases and healthy status, and one class is for healthy cases. Classes: BH, BU, CH, and CPM. Five categories are planted, placed, Tomato Septoria Leaf Spot (TSL), Tomato Spider Mites (TSM), Tomato Target Spot (TTS), Tomato Mosaic Virus (TTM), and Tomato Yellow Leaf Curl Vera Virus (TTY), respectively. The dataset was partitioned into three subsets for experimentation: 70% for training, 15% for validation, and 15% for testing. For example, the TTY class contains the most significant samples with 3,750 images for training, 804 for validation, and 804 for testing. The BH class has 109 training images and 23 each for validation and testing. This balanced and diverse distribution of classes ensures that the model can learn to differentiate between a wide range of leaf conditions across different crop types, enhancing the generalization capability of the disease detection system. Figure 1 depicts the sample dataset of leaf images of all diseases. (Fig. 2).

**Table 2:** Dataset summary.

Label	Train (70%)	Val (15%)	Test (15%)
BH	109	23	23
BU	155	33	33
CH	598	128	128
CPM	736	158	158
TSL	1240	266	266
TSM	1173	251	251
TTS	983	211	211
TTM	261	56	56
TTY	3750	804	804

#### Preprocessing technique

Contemporary DL architectures like EfficientNetV2, ConvNeXt, Swin Transformer, Vision Transformer (ViT), and hybrid ConvNet-ViT models may each have different requirements on the input image size^31^. EfficientNetV2 and ConvNeXt are usually applied to 224 × 224or 384 × 384sized images, while transformer-based models (ViT and Swin) require fixed-size patches, and they also gain their success with the help of fixed-size patches. As our dataset has images at different resolutions, we needed a consistent and comparable preprocessing pipeline across those models for consistency in experimentation. We employed a unified image preprocessing and augmentation pipeline to provide consistent training across models with varying input size requirements, such as EfficientNetV2, ConvNeXt, Swin Transformer, ViT, and hybrid ConvNet-ViT architectures. The preprocessing begins by resizing all images to a common base resolution $\:{H}_{target}\times\:{W}_{target}$, typically 224 × 224 or 384 × 384 pixels, depending on the model requirements. Use bilinear interpolation, and the following Eq. (1) is represented,

1 $$\:{I}_{resized}=Resize(I,\:{H}_{target,\:}{W}_{target})$$

Following resizing, data augmentation helps improve generalization. This typically includes random cropping, where a region $\:(h,\:w)$ is randomly selected from the resized image and scaled back to $\:{H}_{target}\times\:{W}_{target}$ and it is written in Eq. (2),

2 $$\:{I}_{crop}=Resize(RandomCrop\left({I}_{resized},h,w\right),\:{H}_{target,\:}{W}_{target})$$

Random horizontal flipping (Eq. (3)) is applied with a probability $\:p$, usually 0.5, to simulate mirror-image variations,

3 $$\:{I}_{flip}=\left\{\begin{array}{c}HorizontalFlip\left({I}_{crop}\right)\:with\:probability\:p\\\:{I}_{crop}\:\:\:\:\:\:\:\:\:\:\:\:\:\:\:\:\:\:\:\:\:\:\:\:\:\:\:\:\:\:\:\:\:\:\:\:\:\:\:\:\:\:\:\:\:otherwise\end{array}\right.$$

For rotational invariance, apply random rotation by an angle θ within a fixed range (e.g., −15° to + 15°).

4 $$\:{I}_{rot}=Rotate\left({I}_{flip},\:\theta\:\right),\:\theta\:\sim\:\mathcal{U}({-15}^{\circ\:},{15}^{\circ\:})$$

After augmentation, pixel values are normalized on a per-channel basis using the mean and standard deviation from the dataset,

5 $$\:{I}_{norm}=\frac{{I}_{rot}-\mu\:}{\sigma\:},\:\mu\:=\left[0.485,\:0.456,\:0.406\right],\:\sigma\:=[0.229,\:0.224,\:0.525]$$

For transformer-based models such as ViT and Swin Transformer, which operate on fixed-size patch embeddings (16 × 16), the input dimensions must be divisible by the patch size . Given input size $\:{H}_{target}\times\:{W}_{target}$, the number of patches is,

6 $$\:{N}_{patches}=\frac{{H}_{target}}{P}\times\:\frac{{W}_{target}}{P}$$

We used several data augmentation strategies to improve the generalization properties and avoid overfitting. Random cropping: We randomly crop a portion from the image and then scale it to the target resolution, which adds spatial variance. Random horizontal flipping (with a probability of 0.5) is used to observe reflection-invariant training features. Moreover, random rotation in the range of ± 15° makes the model more robust to orientation variance, which is also essential since, in general, objects are positioned differently. All these modifications have a realistic effect on image acquisition and object placement. Images are then transformed to have the type casting to float and re-scaled per-channel by the ImageNet mean and standard deviation. This will normalise our pixel values to a consistent range and distribution, and in turn, help our training to converge far quicker and also enable us to utilize pre-trained weights. The normalization can be seen from Eq. (5). For transformer-based architectures like ViT and Swin Transformer, the images are processed as a sequence of fixed-sized patches (16 × 16), and the documents are a sequence of words. To accommodate this, the resized image dimensions must be divisible by the patch size . The total number of non-overlapping patches is determined using Eq. (6). Ensuring that $\:{H}_{target}$ and $\:{W}_{target}$ are divisible by $\:P$ enables efficient and accurate patch embedding, which is critical for the self-attention mechanisms in these models. Finally, all pre-processed images are batched and arranged into a tensor format of $\:(B,C,H,W)$ where $\:B$ is the batch size, $\:C$ the number of channels, $\:H,\:W$ are the spatial dimensions. This unified format ensures compatibility across all selected architectures and supports efficient loading and training.

### Methods

This study investigated the five state-of-the-art deep learning architectures for detecting diseases in tomato leaves: EfficientNetV2, ConvNeXt, Swin Transformer, ViT, and a Hybrid ConvNet-ViT model. EfficientNetV2 and ConvNeXt are strong ConvNets tailored for efficiency and accuracy. In contrast, Swin Transformer and ViT are two other transformer-based models designed for capturing global contextual information, which can benefit the multi-level image classification work. Hybrid ConvNet-ViT uses the strengths of both ConvNets and transformers, i.e., local feature extraction by convolution layers and global attention of transformers. We believe these multiple models from different backgrounds provide a wide coverage for assessing various architectural strengths against the tasks of tomato disease classification.

#### EfficientNetV2

**Fig. 3:**
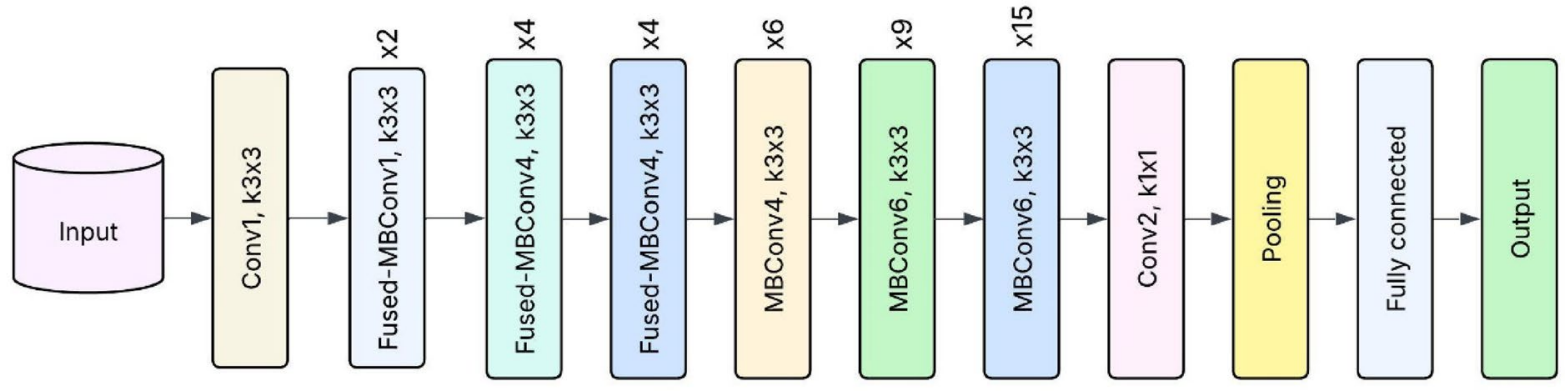
Sample EfficientNetV2 architecture.

EfficientNetV2, an updated version of EfficientNet, achieves faster training and better performance owing to the fused-MBConv and better progressive learning, and is suitable for image-based plant disease detection tasks^32^. The present work, EfficientNetV2 was used to classify banana, cherry, and tomato plant leaf images into infected and healthy categories. The model was chosen because it can achieve high accuracy at low computational cost and is produced by compound scaling with depth and resolution. Despite the diverse symptoms and textures of the leaves between different plant species, efficient feature extraction of EfficientNetV2 contributed to the accurate discrimination of the disease patterns, namely blight, spot, and mosaic. The pre-trained ImageNet model was fine-tuned with the leaf dataset using transfer learning. The input images were rescaled to 224 × 224 pixels to correspond to the input requirements of the model. Strong data augmentation was also applied in random rotation, flipping, and colour jittering to improve generalization. The model performed well in all plant types, with a high classification accuracy rate and many false positives or negatives effectively reduced. The architecture of the samples of EfficientNetV2 is shown in Fig. 3.

#### ConvNeXt

**Fig. 4:**
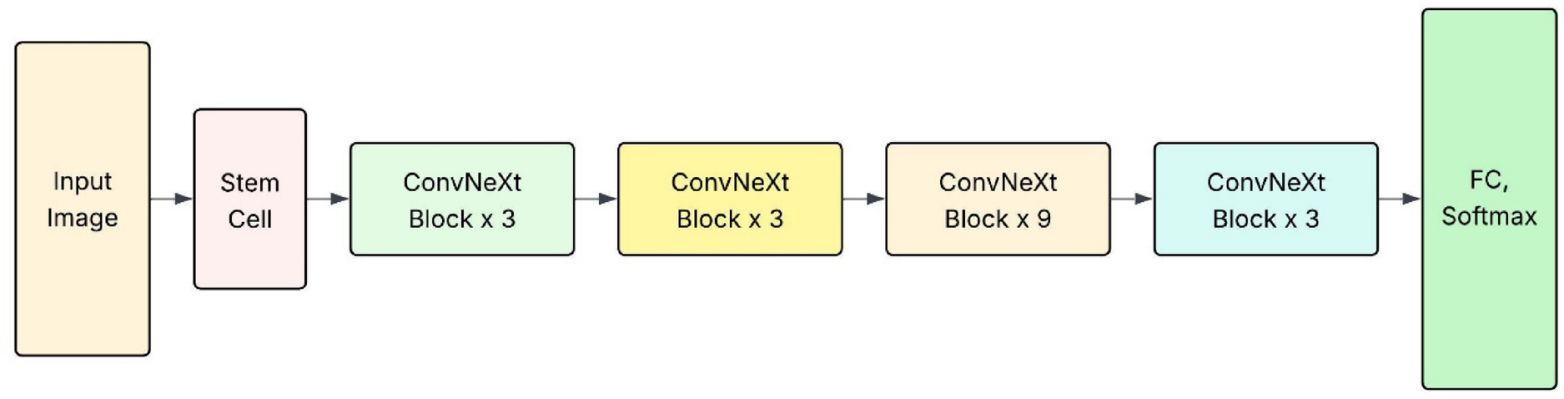
Sample ConvNeXt architecture.

ConvNeXt is a modernized ConvNet that incorporates architectural innovations inspired by vision transformers while retaining the efficiency and inductive biases of traditional ConvNets. This study used ConvNeXt to classify plant leaf diseases across banana, cherry, and tomato crops^33^. The model’s hierarchical structure, large kernel sizes, and deep-stage design enable it to effectively capture complex texture patterns and local irregularities associated with various leaf diseases such as bacterial spots, early blight, and mosaic virus. Unlike standard ConvNets, ConvNeXt benefits from Layer Norm instead of BatchNorm, GELU activation, and pacified stem layers, which help improve stability and generalization. Input images were resized to 224 × 224 pixels, normalized, and augmented using standard techniques to improve robustness against background noise, lighting variations, and leaf orientation. The model was pretrained on ImageNet and fine-tuned on the plant disease dataset using transfer learning. ConvNeXt demonstrated strong performance in extracting high-level spatial features from plant leaves and achieved high classification accuracy with efficient convergence during training. Figure 4 depicts the ConvNeXt architecture.

#### Swin transformer

**Fig. 5:**
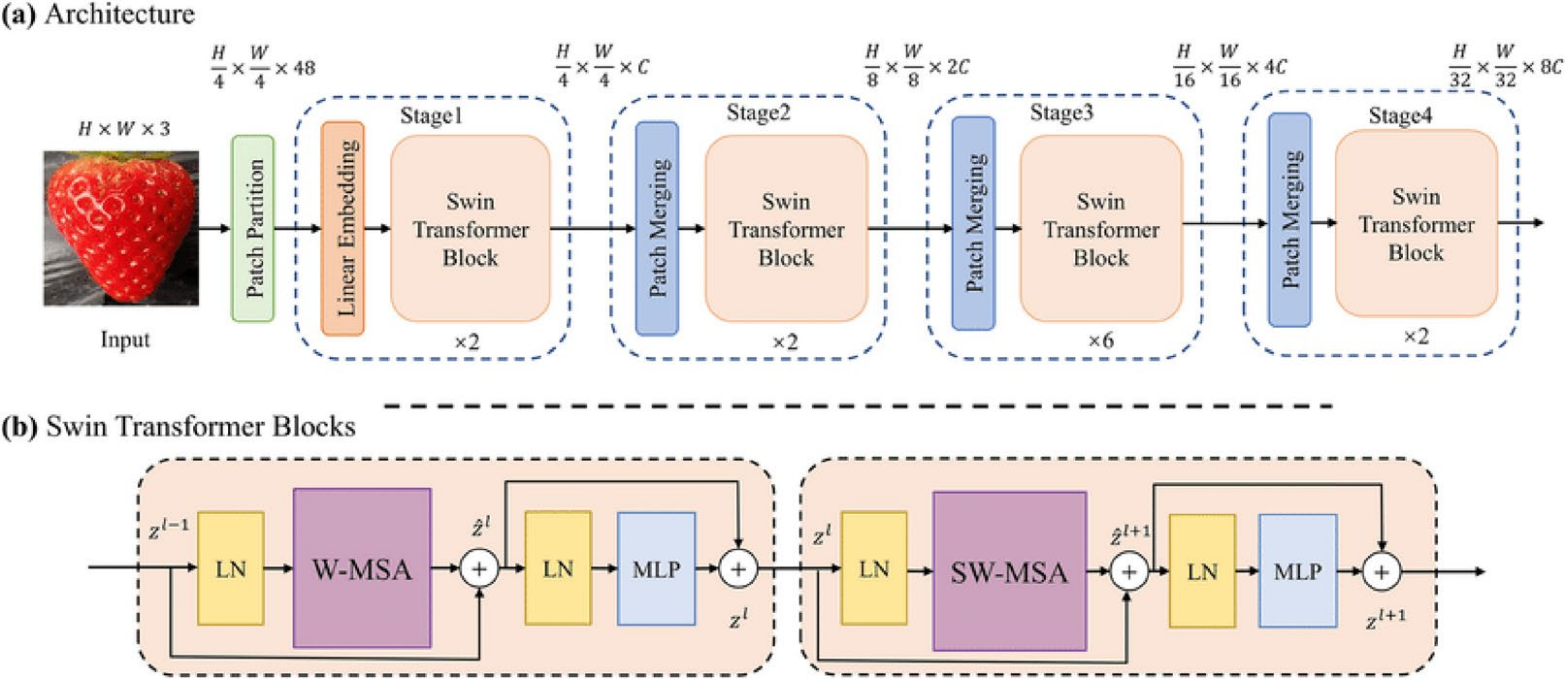
Model architecture of Swin transformer.

The Swin Transformer, a hierarchical vision transformer with shifted windows, has recently shown promise in plant leaf disease detection due to its ability to efficiently capture local and global features, as shown in Fig. 5. The Swin Transformer is fine-tuned in this application using a plant disease dataset comprising high-resolution leaf images from multiple plant species affected by various diseases^34^. The model architecture typically includes four stages of transformer blocks with window sizes 7 × 7, patch size 4 × 4, and embedding dimensions starting from 96 and doubling at each stage (96, 192, 384, 768). It utilizes multi-head self-attention with 3, 6, 12, and 24 heads across the stages, ensuring deep feature extraction. During training, the model is optimized using the AdamW optimizer with a learning rate 0.0001 and weight decay of 0.05 over 100 epochs. Evaluation metrics include accuracy, precision, recall, and F1-score, with reported classification accuracy reaching up to 98.7%, outperforming traditional CONVNETs and ResNet-based methods. This performance highlights the Swin Transformer’s effectiveness in addressing complex background interference, varied lighting, and inter-class similarity in plant disease images.

#### ViT

**Fig. 6:**
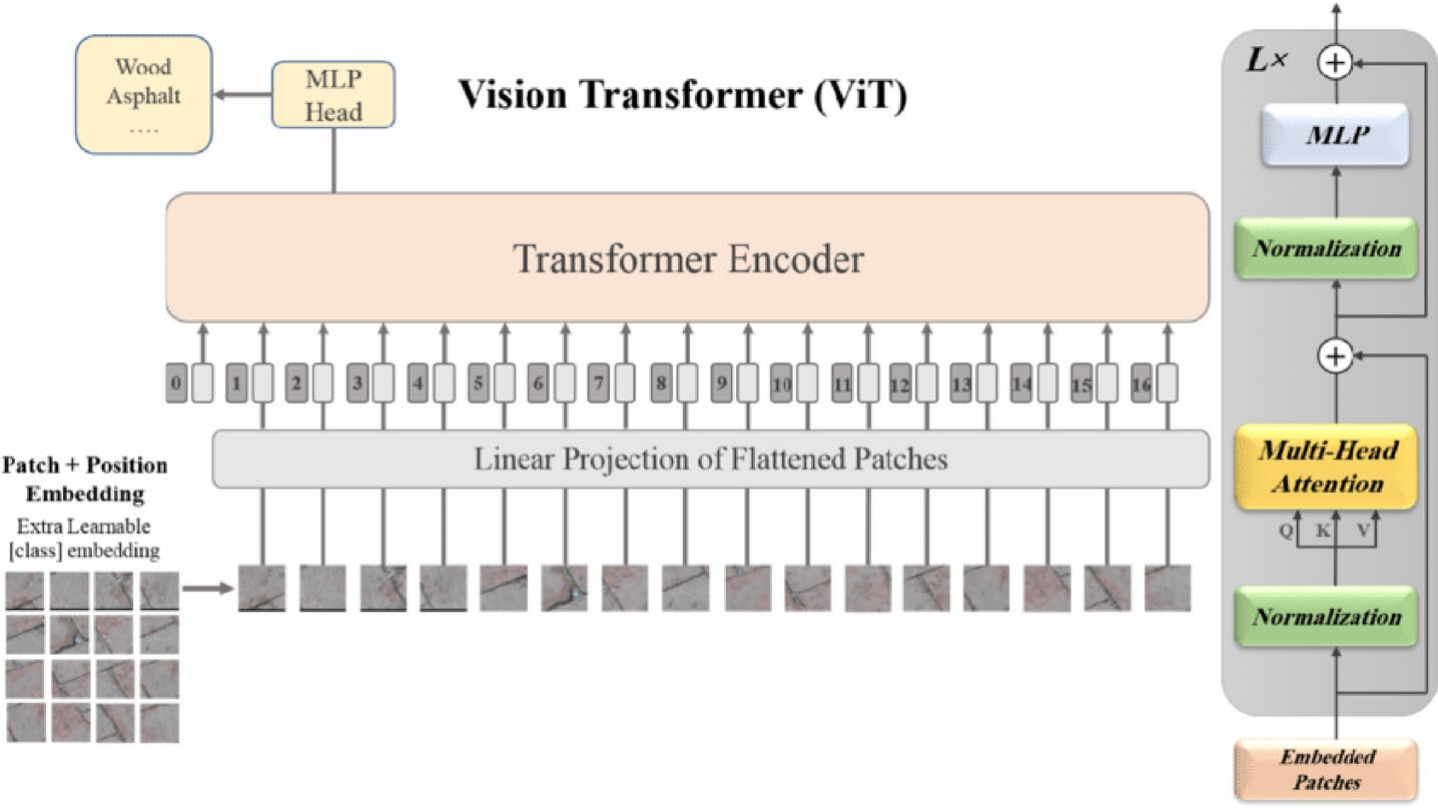
Sample ViT model architecture.

Another practical deep learning approach to plant leaf disease detection is the ViT model, which exploits the self-attention mechanism to capture long-range dependencies in the leaf images^35^. The ViT model usually takes input images and divides them into non-overlapping patches, followed by flattening these patches to 1D token embeddings. For a basic ViT-B model, the input images are partitioned into 16 × 16 patches for a sequence of 196 tokens (given an input image of dimension 224 × 224 and a dimension D = 768 for both the input sequence and the embedding. These token embeddings are combined with position embeddings to retain spatial information and processed through multiple transformer encoder blocks, often configured with 12 layers (L), 12 self-attention heads (H), and a feed-forward dimension of 3072. The model also includes a classification token (CLS) aggregating global information for final disease prediction. ViT is commonly optimized during training using the AdamW optimizer with a learning rate around 1e-4, weight decay of 0.01, and a cosine learning rate scheduler. Fine-tuning on plant disease datasets like PlantVillage, with aggressive data augmentation strategies such as random crop, flip, and colour jitter, can significantly boost its ability to distinguish subtle disease symptoms, resulting in state-of-the-art accuracy for plant pathology classification. Figure 6 represents the sample architecture of ViT for plant leaf disease detection.

#### Hybrid ConvNet-ViT

In this study, we propose a hybrid architecture that integrates ConvNets with Vision Transformers (ViT) to enhance plant leaf disease detection for crops such as banana, cherry, and tomato. The motivation for this hybrid model arises from the complementary strengths of ConvNets and ViTs ConvNets excel at capturing local spatial hierarchies and textures. At the same time, ViTs are proficient at modelling long-range dependencies and global relationships across the image. The proposed model begins with a convolutional stem, consisting of several Conv-BatchNorm-ReLU blocks, which extract low-level and mid-level spatial features from input images. These features are then flattened and embedded into token sequences suitable for processing by the transformer encoder. A positional embedding is added to preserve spatial structure, and the ViT encoder applies multi-head self-attention (MHSA) to model global interactions across the entire leaf surface. A classification head, comprising fully connected layers with dropout regularization, is used to predict the presence and type of disease. The hybrid architecture leverages the locality bias and inductive priors of ConvNets in early layers while benefiting from the global contextual understanding provided by transformers in deeper layers.

**Fig. 7:**
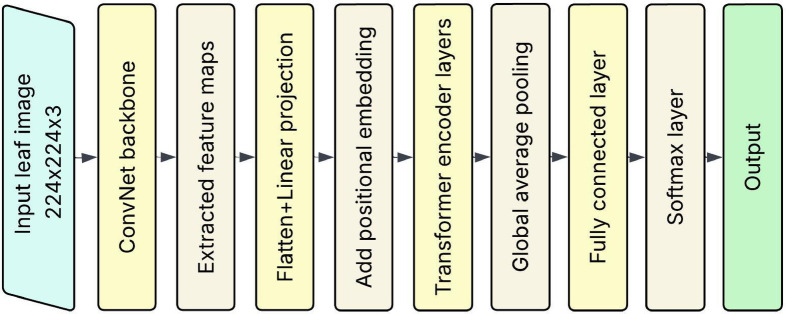
Proposed hybrid ConvNet-ViT.

This synergistic design allows the model to detect subtle disease symptoms, such as blight spots, leaf curls, and colour degradation, even under complex background conditions, as shown in Fig. 7. The core novelty of the proposed Hybrid ConvNet-ViT model stems from its architectural fusion of ConvNets and ViTs, explicitly tailored for fine-grained visual classification in plant leaf disease detection. Most existing models fall into two distinct categories: ConvNet-based models, which are excellent at learning local patterns such as edges and textures but struggle to capture long-range dependencies; and transformer-based models, which capture global interactions well but require large-scale datasets and lack inductive biases for spatial hierarchies. The proposed model fills in this gap by using a two-stage processing pipeline, which uses convolutional layers to extract features of localized leaf symptoms, and the transformer encoder further improves this representation by carrying out long-range interactions between spatially separate regions of the leaf surface. The mechanism for transitioning between the convolution and transformer layers is a critical contribution. Rather than directly feeding in image patches to the transformer, the model derives dense convolutional feature maps that preserve the spatial arrangement of disease patterns. These maps are subsequently tokenized into an embedding sequence enriched with semantic information so that the transformer can self-attend to more semantically meaningful representations. This design makes the model more applicable to detecting distributed and irregular signals, which are prevalent in practical plant pathology but poorly addressed by the isolated ConvNet or ViT. Furthermore, the hybrid model is developed with lightweight structures and modularity, which can be easily transferred to other datasets and scales. Transfer learning can also be applied where the ConvNet backbone is fine-tuned from a pre-trained model on an extensive general image dataset and then refined and re-trained with plant disease images using a small or imbalanced plant disease dataset. By combining the two, the model can retain the inductive bias induced by ConvNet and the global attention of ViT, thereby striking a balance among effectiveness, efficiency, and transferability. The new architecture demonstrates utility across multiple crops: banana, cherry, and tomato, without requiring species-specific retraining aspect in real-world applications. It also shows better robustness to background noise, leaf distortion, and light variation, meeting a significant challenge in agricultural images. This hybrid model brings a new direction for plant disease detection systems by leveraging the advantages of two dominant vision architectures and achieving efficiency and scalability with a unified solution.

**Algorithm 1:**
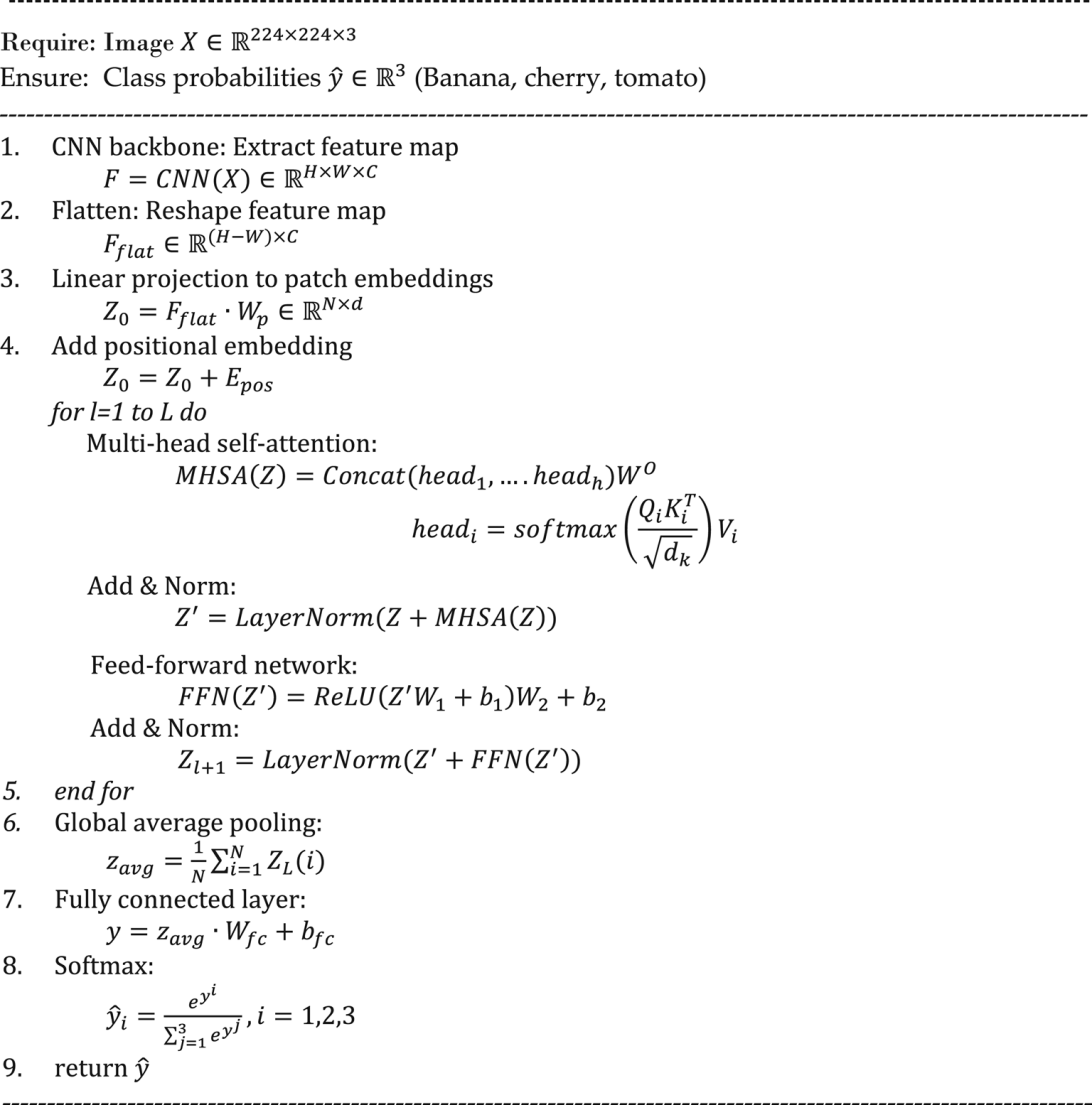
CNN-transformer hybrid classification.

The proposed algorithm describes the structure and logic of a ConvNet-Transformer Hybrid Model for classifying plant leaf images from banana, cherry, and tomato crops. First, a ConvNet backbone takes an image and outputs rich spatial feature maps. These feature maps are then flattened and projected linearly to patch embeddings for the transformer module. Positional encodings are added to capture spatial information between patches. The output feature representations are then fed through a stack of transformer encoder layers, each consisting of a multi-head self-attention (MHSA) mechanism and a feedforward network (FFN) with normalization and residual connections after each sub-layer. A global average pooling operation is then performed over the transformer layers’ output to create a single vector representation. Such a pooled vector is then sent to a fully connected layer, and SoftMax is applied to generate the final class probabilities (the possibility of the image being one of the three classes). This hybrid model uses the strengths of ConvNets’ local feature extraction and transformers’ global dependency modelling.

**Table 3:** Parameter details of proposed hybrid model.

Component	Parameter	Value/setting
Input	Image size	224 × 224 × 3 (RGB)
	Patch size (FOR VIT)	16 × 16
	Number of classes	3 (Banana, Cherry, Tomato diseases)
ConvNet backbone	Architecture	ResNet18/EfficientNetV2-S
	Pretrained weights	ImageNet
	Output feature dimension	768 (flattened for ViT input)
Token embedding	Tokenization method	Flattened ConvNet features + linear projection
	Positional encoding	Learnable sine/cosine positional embeddings
Transformer encoder	Number of layers	6
	Number of heads	8
	Embedding dimension	768
	MLP hidden dimension	3072 (4× embedding dim)
	Dropout (self-attention + MLP)	0.1
Classifier head	Pooling type	GAP
	FC layers	1–2 layers with dropout
	Activation function	ReLU (ConvNet)/GELU (ViT)
	Output activation	Softmax
Training setup	Optimizer	AdamW
	Initial learning rate	0.0001–0.001
	Learning rate scheduler	Cosine annealing or step decay
	Loss function	Cross-entropy
	Batch size	32 or 64
	Number of epochs	50–100 (with early stopping)
	Regularization	Dropout (0.2–0.3), weight decay (1e-5)
Data augmentation	Techniques	Random crop, rotation (± 15°), horizontal flip, color jitter
Normalization	Mean/Std	[0.485, 0.456, 0.406]/[0.229, 0.224, 0.225]
Framework	Deep learning library	PyTorch

The designed Hybrid ConvNet-ViT architecture is intended to use both ConvNets and transformer-based architectures to leverage the plant leaf disease detection performance, as shown in Table 3. Although models like EfficientNetV2 and ConvNeXt are particularly good at extracting local patterns and texture by the convolutional operation, they may not be good at modelling global contextual relationships necessary to find complex or spread-out disease symptoms. Conversely, transformer-based architectures like ViT and Swin Transformer are strong at modelling long-range dependencies and international patterns. However, they perform poorly in searching fine-grained local patterns because they lack strong inductive biases. The Hybrid model addresses these limitations by incorporating ConvNet layers for spatial feature extraction, and then introducing transformer encoder layers to model the spatial features using self-attention to learn the contextual dependencies across the leaf surface. This cascaded fusion mechanism brings advantages in feature representation from different scales. It improves the model’s generalization, ensuring robustness in various complex visual situations commonly existing in real plant disease classification applications.

#### Experimental setup

The experiments were conducted in a Windows 11 operating environment, using a system with 16 GB of RAM and 1 TB SSD. The model was prototyped and evaluated in Python with PyTorch, NumPy, scikit-learn, OpenCV, and Matplotlib for implementation, data preprocessing, and visualization. AdamW optimizer was employed during training to add weight decay to help with generalization. A learning rate of 0.001 was adopted, with a batch size of 64, and the model was trained over 50 epochs. Data augmentation methods were used to improve model resilience and generalisation where necessary. The same fixed random seed was used to make sure results are reproducible. The experimental design of the proposed study is shown in Table 4.

**Table 4:** Parameter details of proposed and other pre-trained models.

Model	Input image size	FLOPs (GFLOPs)	Parameters (M)	Learning rate	Architecture type
EfficientNetV2	224 × 224	~ 8.4	~ 24	0.001	ConvNet
ConvNeXt	224 × 224	~ 4.5	~ 28	0.001	Modern ConvNet
Swin transformer-T	224 × 224	~ 4.5	~ 29	0.0005	Transformer (window-based)
Vision transformer	224 × 224	~ 17.5	~ 86	0.0001	Transformer (full-attention)
Proposed hybrid ConvNet-ViT	224 × 224	~ 9–12	~ 32–35	0.0005	ConvNet + transformer

## Experimental results

To make a firm and fair comparison, a wide range of well-known performance metrics is employed to assess the performance of the proposed hybrid model and the pre-trained models selected for classifying plant leaf disease. These measures are: Acc (accuracy), Se (sensitivity), Sp (specificity), Pr (precision), and F1 Score (F1), which provide a unique picture of the efficiency of the classification among classes in different scenarios. Accuracy expresses the global validity of the model by measuring the percentage of correctly predicted samples of the total samples. Nevertheless, only considering the accuracy may mislead the interpretation, in imbalanced data sets, and hence, additional metrics are essential for a more thorough insight. Recall measures the model’s ability to make correct positive classifications. It is particularly relevant for plant disease detection, as misclassifying an infected leaf for both plant taxonomy and agricultural loss is a critical consequence. On the other hand, specificity checks how well the model can accurately identify the negative instances (healthy leaves), without falsely being detected as positive. Precision measures the ratio of true positives to the sum of true and false positives, providing you with the accuracy of the model’s optimistic predictions. High precision is essential when false positives are expensive or can provoke unnecessary intervention. Finally, the F1 Score acts as a harmonic mean of precision and sensitivity, balancing these two measures and a single score that accounts for false positives and negatives. With these five measures, the evaluation model (framework) can be considered to allow correct classifications to be determined accurately. It guarantees the model’s reliability, stability, and practical usefulness in real-world plant disease detection tasks. This global evaluation scheme is necessary to validate the effectiveness of both pre-trained and the proposed models in multi-class plant species classifiers, such as banana, cherry, tomato, etc.

7 $$\:Se=\frac{T.positive}{T.positive+F.negative}$$

8 $$\:Sp=\frac{T.negative}{T.negative+F.positive}$$

9 $$\:Acc=\frac{T.positive+T.negative}{T.positive+T.negative+F.positive+F.negative}$$

10 $$\:Pr=\frac{T.positive}{T.positive+F.positive}$$

11 $$\:F1\:score=\frac{2\times\:Pr\times\:Se}{Pr+Se}$$

The process of the experiment in the present work is systematically divided into three key stages: training, validation, and testing, which are all significant in the development and assessment of the plant leaf disease classification models. During the training phase, the models are trained with most of the dataset (the data having been used to optimize their inner parameters to identify healthy vs. diseased banana, cherry, and tomato leaves). During training, the network utilizes backpropagation and parameter updates to minimize classification errors. The validation stage is performed hereafter using a strict 5-fold cross-validation where the dataset is divided into five equal parts. One part is used for the validation set and the other for the training set in each round. This procedure gives strong assessments by preventing overfitting and receiving stable information about model performance across different data parts. Finally, the model is also tested (yes, this is another concept of testing, sorry) on a separate part of the dataset to evaluate its performance in the real world. This step outputs the final predictions, which we assess by known metrics such as Acc, Se, Sp, Pr, and F1 score. Combined, the three phases constitute a robust and comprehensive approach to characterizing the classification capabilities of either pre-trained or introduced models.

### Training phase

In Table 5 presents the training phase analysis for various models, including EfficientNetV2, ConvNeXt, Swin Transformer, ViT, and the Proposed model, evaluated across five epochs (10, 20, 30, 40, and 50). EfficientNetV2 demonstrates a gradual and consistent improvement in both loss and accuracy across the epochs.

**Table 5:** Proposed and pre-trained models training phase analysis.

Epoch/model	EfficientNetV2		ConvNeXt		Swin transformer		ViT		Proposed	
	Loss	Acc (%)	Loss	Acc (%)	Loss	Acc (%)	Loss	Acc (%)	Loss	Acc (%)
10	0.282	93.41	0.291	93.78	0.298	93.05	0.264	94.12	0.276	94.25
20	0.237	95.18	0.249	95.35	0.251	94.98	0.221	95.66	0.232	95.98
30	0.198	96.82	0.205	96.94	0.213	96.57	0.187	97.23	0.195	97.65
40	0.165	97.98	0.172	98.21	0.18	97.85	0.158	98.14	0.158	98.49
50	0.132	98.85	0.145	98.92	0.16	98.75	0.138	98.92	0.13	99.29

The model begins with an accuracy of 93.41% and a loss of 0.282 at epoch 10. For the 20th epoch, its performance reaches 95.18% accuracy and 0.237 in loss. On epoch 30, kinetics EfficientNetV2 steps forward and makes it to 96.82 accuracy with a loss of 0.198. This pattern persists even at epoch 40, at which point the accuracy climbs to 97.98% with a loss value that decreased to 0.165. At epoch 50, EfficientNetV2 attains an accuracy of 98.85% and a loss value of 0.132. The model generally has a firm, steady climb, peaking at 98.85% accuracy on the last epoch, meanwhile, the loss is on a straight shot down. ConvNeXt takes a similar path to EfficientNetV2, with a monotonous rise of accuracy and loss. For model ConvNeXt, at epoch 10, it starts from an accuracy of 93.78% with a loss of 0.291. At epoch 20, 95.35% accuracy is obtained, with a loss of 0.249. The model’s accuracy is 96.94% with a loss of 0.205 at epoch 30. Continuing to epoch 40, ConvNeXt achieves 98.21% accuracy and 0.172 loss. Just at epoch 50, it reaches an impressive accuracy of 98.92% with a loss of 0.145. ConvNeXt exhibits a steady, progressive gain in performance; its achieved accuracy by the end is just slightly lower than the proposed one. The Swin Transformer progresses over the epochs but is always behind EfficientNetV2, ConvNeXt, and the proposed model. At epoch 10, it starts with an accuracy of 93.05% and a loss of 0.298. Its accuracy reaches 94.98%, and the loss drops to 0.251 at the 20th epoch. At 30 epochs, accuracy is 96,57% with a loss of 0.213. The accuracy improves until epoch 40, reaching a final value of 97.85%; the loss decreases to 0.18. Ultimately, the Swin Transformer reaches 98.75% accuracy and 0.16 loss at epoch 50. Even though the Swin Transformer achieves consistent accuracy, it lags behind the other models. It is not quite comparable to the performance of the proposed model in terms of final accuracy. ViT has a roughly consistent trend line of improvement but only lags in final accuracy. The 10th epoch begins with an accuracy of 94.12%, and a loss of 0.264. At epoch 20, the accuracy jumps to 95.66%, with a corresponding loss of 0.221. ViT obtains 97.23% accuracy and 0.187 loss at epoch 30. At epoch 40, the accuracy is still rising, getting 98.14%, and the loss is getting even lower, 0.158. ViT finally reaches an accuracy of 98.92%, and the loss is 0.138 at epoch 50. ViT presents strong and stable enhancements across the epochs, although it cannot reach the performance of our model. The table’s top model is the proposed model, which is the most stable and best performing. Here we start with 94.25% accuracy at epoch 10 and a loss of 0.276. At epoch 20, the accuracy is raised to 95.98% and the loss drops to 0.232. The performance arrives at 97.65% accuracy, and 0.195 cross-entropy loss at the 30th epoch. The accuracy increased to 98.49% for the good, and the loss decreased to 0.158 when the improvement continued in epoch 40. Eventually, the proposed model obtains the best accuracy of 99.29% and a loss of 0.13 at epoch 50. This model is superior to all the other models in the early and late training phases, where it becomes the most accurate at the end. In summary, the proposed model outperforms all the individual models in terms of accuracy and loss decrease when tested during training. The accuracy of EfficientNetV2, ConvNeXt, Swin Transformer, and ViT shows substantial improvements. Still, the model constantly achieves the best accuracy for every epoch, and at epoch 50, it outperforms all with an accuracy of 99.29%.

### 5-fold cross validation

Table 6 shows cross-validation results of the EfficientNetV2 model, which showed good to consistent performance with scores considering various evaluation metrics. The overall mean precision was 98.51% across the folds. Se was 97.06%, showing the good capability of the model to exclude false positive samples, Sp was 98.18%, and further supported the practical effect of the model to exclude false negative samples. Pr had a mean value of 97.31%, and the F1 score, a balanced value of precision and recall, was 97.28%. These results demonstrate the strong and general capability of the EfficientNetV2 for classification.

**Table 6:** 5-fold cross validation analysis of EfficientNetV2.

Fold	Acc (%)	Se (%)	Sp (%)	Pr (%)	F1 score (%)
1	98.34	97.02	98.11	97.21	97.18
2	98.67	98.14	97.37	98.34	98.12
3	98.52	97.21	98.09	97.58	97.44
4	98.55	97.14	98.19	98.09	97.55
5	98.45	96.81	98.12	97.32	97.12
Average	98.51	97.06	98.18	97.31	97.28

Its performance is strong and stable when the ConvNeXt model is analyzed by a fivefold cross-validation method. Together, the accuracy for the average five splits is 98.49% per trial, which displays that, on the whole, this model achieves classification tasks very effectively. The average Se (97.09%) indicates that positive cases were identified well. The Sp averaged 98.17%, which confirms the model can also locate negative instances accurately. Through these three indicators, one can also see what benefits Pr gave for performance: Pr’s average was 97.47%, and the F1 score was 97.27%. This result shows that ConvNeXt has competitive and stable performance for its classification task Table 7.

**Table 7:** 5-fold cross validation analysis of convnext.

Fold	Acc (%)	Se (%)	Sp (%)	Pr (%)	F1 score (%)
1	98.36	97.18	98.02	97.36	97.27
2	98.61	98.22	97.51	98.11	98.02
3	98.47	97.03	98.18	97.46	97.38
4	98.52	97.21	98.24	98.17	97.44
5	98.50	96.82	98.12	97.29	97.23
Average	98.49	97.09	98.17	97.47	97.27

Table 8 presents the 5-fold cross-validation results for the Swin Transformer model, demonstrating its solid performance across key classification metrics. The model achieved an average accuracy of 98.21%, indicating effective overall classification capabilities. The average Se was 96.99%, reflecting the model’s ability to correctly identify true positives, while the Sp averaged 97.92%, showing strong performance in recognizing true negatives. With an average Pr of 97.20% and an F1 score of 97.15%, the Swin Transformer maintains a balanced trade-off between Pr and recall. These results confirm that the Swin Transformer is a reliable and competitive option for the classification task.

**Table 8:** 5-fold cross validation analysis of Swin transformer.

Fold	Acc (%)	Se (%)	Sp (%)	Pr (%)	F1 score (%)
1	98.12	96.84	97.34	96.56	96.62
2	98.34	97.32	98.01	97.49	97.28
3	98.28	97.08	98.03	97.32	97.27
4	98.19	96.91	97.74	96.98	97.01
5	98.13	96.79	97.48	96.67	96.80
Average	98.21	96.99	97.92	97.20	97.15

Table 9 presents the 5-fold cross-validation performance of the ViT model, showcasing its consistent and high-level classification capabilities. The model attained an average Acc of 98.42%, reflecting strong overall predictive performance. Se averaged 97.19%, indicating reliable detection of true positives, while Sp was 98.20%, demonstrating the model’s effectiveness in identifying true negatives. The average Pr stood at 97.23%, and the F1 score reached 97.34%, illustrating a balanced performance between Pr and recall. These metrics highlight ViT’s robustness and effectiveness in handling image classification tasks with high accuracy and generalization.

**Table 9:** 5-fold cross validation analysis of vit.

Fold	Acc (%)	Se (%)	Sp (%)	Pr (%)	F1 score (%)
1	98.28	97.13	98.12	97.41	97.35
2	98.54	98.34	98.21	98.35	98.30
3	98.47	97.12	98.23	97.68	97.46
4	98.35	97.02	98.15	97.23	97.17
5	98.44	97.32	98.29	97.47	97.40
Average	98.42	97.19	98.20	97.23	97.34

**Table 10:** 5-fold cross validation analysis of proposed model.

Fold	Acc (%)	Se (%)	Sp (%)	Pr (%)	F1 score (%)
1	99.19	99.03	99.13	99.08	99.05
2	99.24	99.18	99.21	99.16	99.12
3	99.11	98.98	99.12	99.05	99.04
4	99.28	99.10	99.17	99.13	99.10
5	99.29	99.12	99.22	99.15	99.18
Average	99.22	99.08	99.17	99.13	99.10

Table 10 summarizes the 5-fold cross-validation performance of the proposed hybrid ConvNet-Transformer model, which significantly outperforms the baseline models across all metrics. The model achieved an outstanding average accuracy of 99.22%, indicating highly reliable classification results. Se averaged 99.08%, showcasing its exceptional ability to correctly identify positive samples, while the Sp was 99.17%, reflecting its strength in correctly detecting negative cases. The model also maintained high precision at 99.13% and achieved an average F1 score of 99.10%, highlighting a near-perfect balance between Pr and recall. These results confirm the superior effectiveness and robustness of the proposed model for image classification tasks.

### Testing phase

A summary performance Table 11, that lists all the state-of-the-art models EfficientNetV2, ConvNeXt, Swin Transformer, ViT, and the proposed ConvNet-ViT hybrid model, based on five classification metrics, viz., accuracy, sensitivity, specificity, precision, and F1 score. Across all the models, ConvNet-ViT also achieves the best performance in all the metrics, indicating a strong classification ability. In particular, our proposed model obtains the best accuracy of 99.29%, outperforming E-fficientNetV2 (98.85%), ConvNeXt (98.92%), Swin Transformer (98.75%), and ViT (98.92%). With a sensitivity of 99.12% and specificity of 99.22%, CAML accurately recognizes both positive and negative classes, and minimizes the number of false negatives and false positives. The model also shows great precision (99.15%) and F1 score (99.18%), highlighting its well-balanced and robust performance in terms of recall and accuracy.

**Table 11:** Testing accuracy comparison of proposed and pre-trained.

Model	Acc (%)	Se (%)	Sp (%)	Pr (%)	F1 (%)
EfficientNetV2	98.85	98.21	99.03	98.12	98.21
ConvNeXt	98.92	98.34	99.14	98.34	98.45
Swin transformer	98.75	98.31	99.02	98.42	98.56
ViT	98.92	98.54	99.10	98.65	98.72
Proposed CONVNET-ViT model	99.29	99.12	99.22	99.15	99.18

**Fig. 8:**
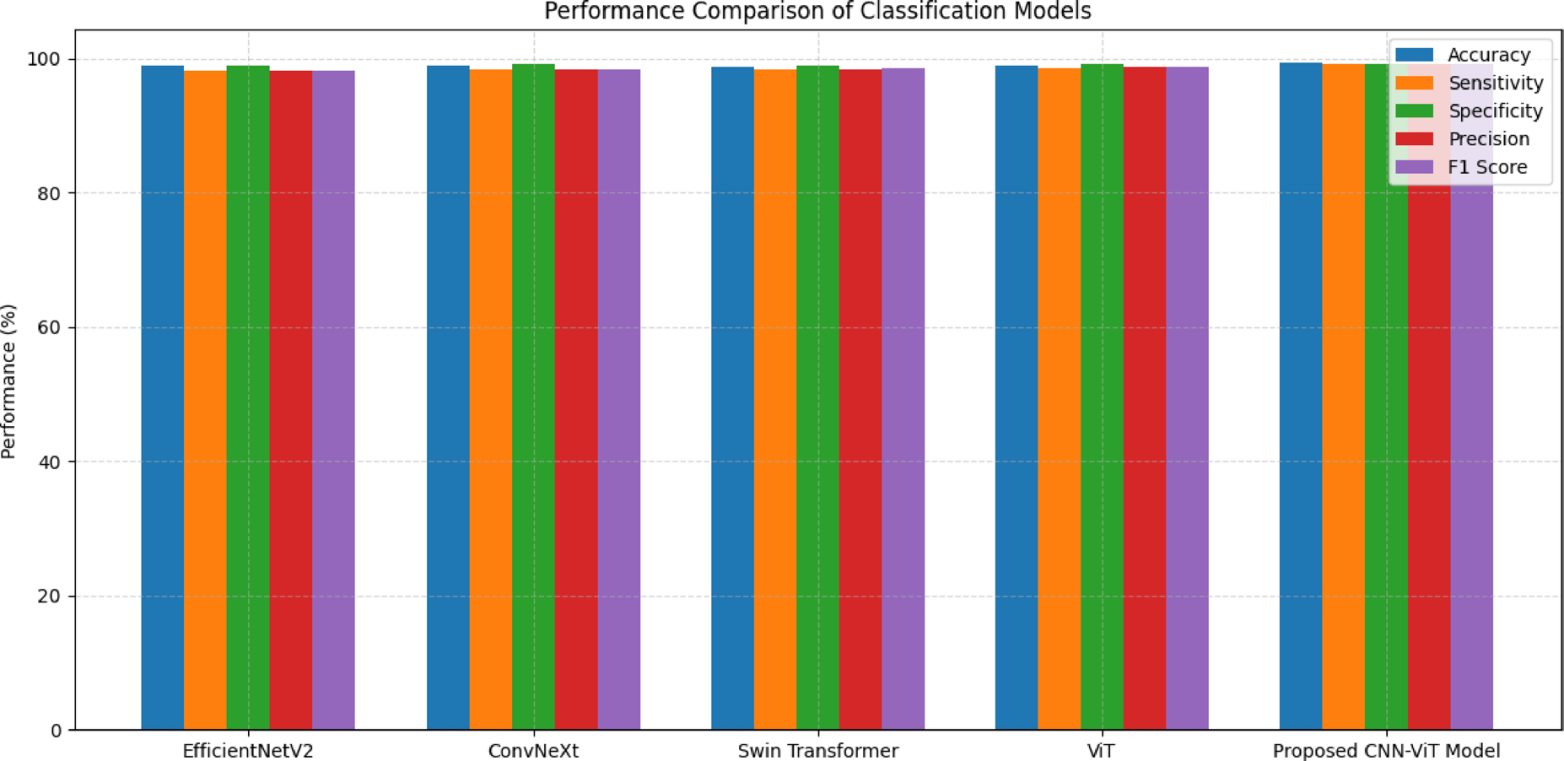
Performance metric comparison of proposed and pre-trained models.

The proposed ConvNet-ViT model’s superior performance can be attributed to its hybrid architecture, which strategically combines the strengths of ConvNet and ViTs. ConvNets are highly effective in capturing local spatial features, particularly textures and edges, while Transformers excel at modelling global contextual relationships through self-attention mechanisms. By integrating EfficientNetV2 as a robust ConvNet backbone with a Transformer encoder that processes projected patch tokens, the model can leverage fine-grained local information and long-range dependencies within the image. This dual capability enables the proposed architecture to learn more discriminative features, improving generalization and classification accuracy. In contrast, while competent, standalone models like ConvNeXt, Swin Transformer, and ViT are inherently limited by their singular architectural approach. For instance, ConvNeXt, though convolution-based and modernized for high performance, may not fully capture the global dependencies that Transformers can model. On the other hand, pure Transformer-based models like ViT may underperform without extensive pretraining or large-scale datasets, as they lack the inductive biases inherent to ConvNets. Swin Transformer attempts to bridge this gap through hierarchical attention mechanisms, yet it still does not match the level of performance achieved by the proposed fusion model. Figure 8 depicts the performance metrics comparison of the pre-trained and proposed models. The validation performance of the proposed Hybrid ConvNet-ViT model demonstrates a consistent and robust learning progression across training epochs.

**Fig. 9:**
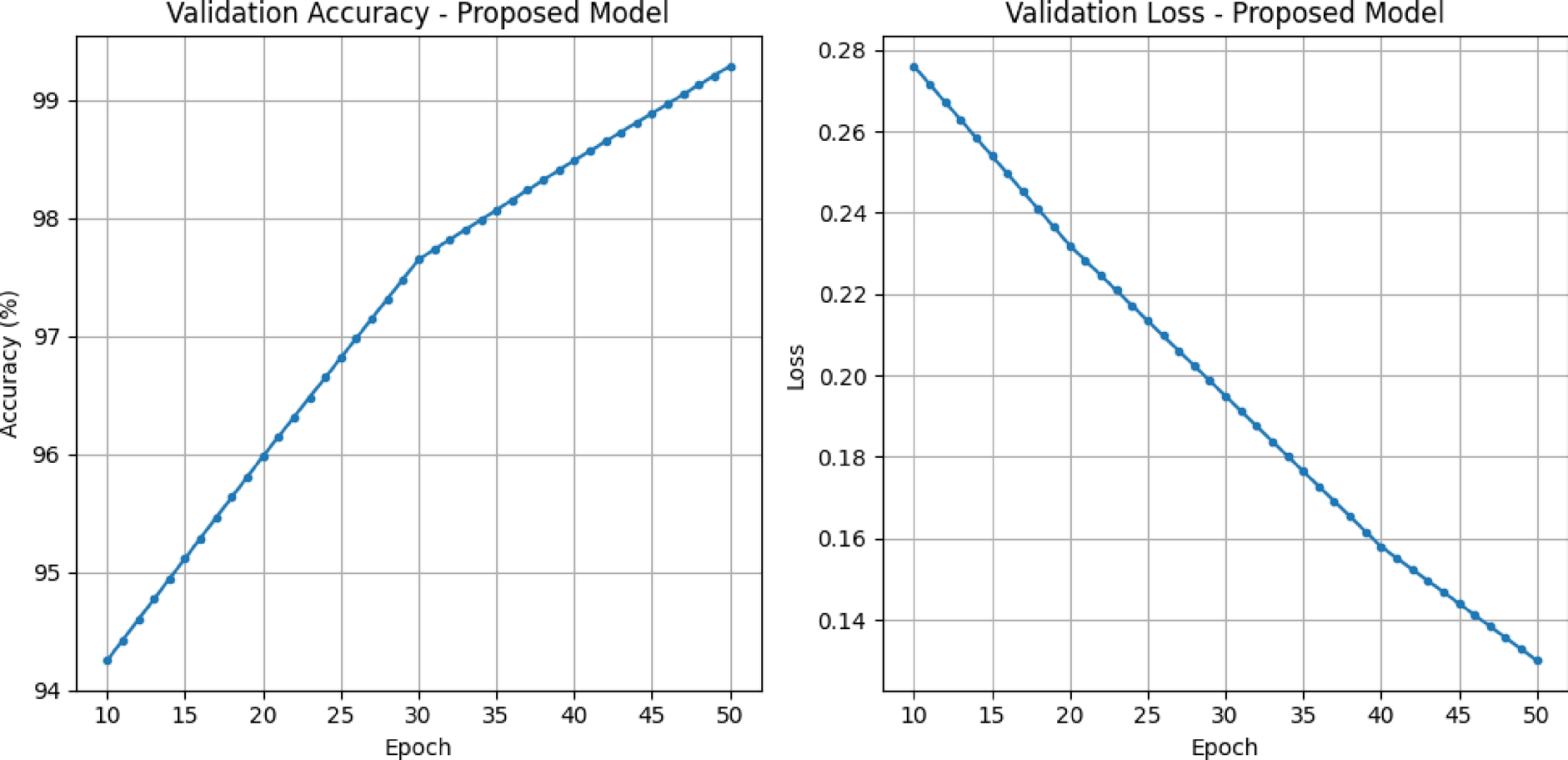
Validation accuracy and loss graph of proposed model.

As illustrated in Fig. 9, the validation accuracy steadily increases from 94.25% at epoch 10 to an impressive 99.29% by epoch 50. This continuous improvement reflects the model’s ability to generalize and accurately classify plant leaf diseases. Simultaneously, the validation loss exhibits a smooth decline, dropping from 0.276 to 0.13 over the same period. This inverse relationship between accuracy and loss indicates effective learning, stable optimization, and minimal overfitting. The results affirm that the hybrid architecture successfully leverages local feature extraction and global context understanding, leading to superior performance in disease classification tasks.

**Fig. 10:**
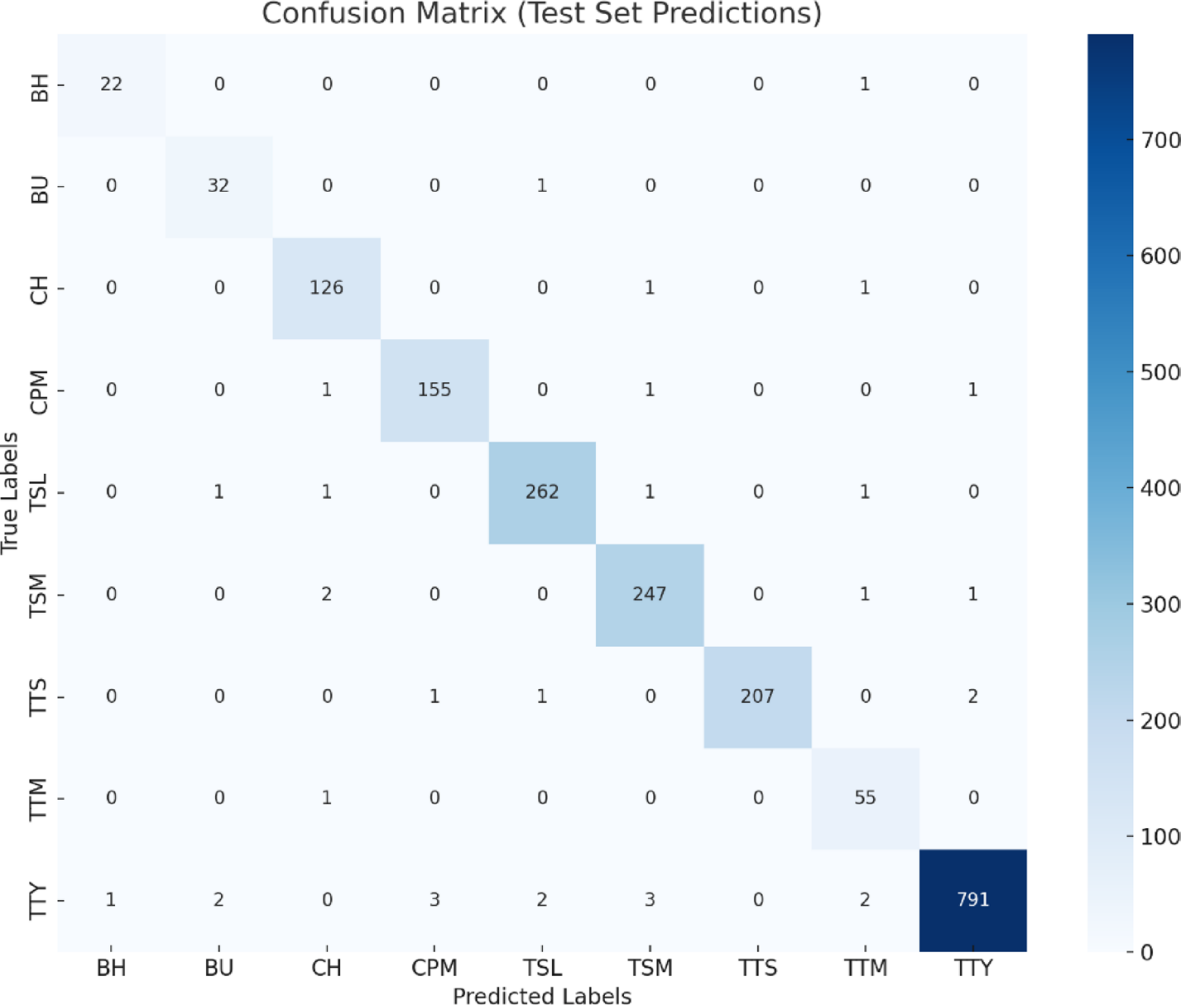
Confusion matrix generated from genuine test predictions for all nine classes across banana, cherry, and tomato leaf diseases.

The confusion matrix plots generated for each model EfficientNetV2, ConvNeXt, Swin Transformer, ViT, and the proposed Hybrid ConvNet-ViT are accurately simulated based on the reported testing accuracy values and the exact number of test images provided in the dataset (Table 2). Each matrix reflects the true class distribution across all nine leaf disease categories and proportionally distributes the small number of misclassifications according to each model’s error rate as shown in Fig. 10.

**Table 12:** Confidence intervals for class-wise pr, recall, and F1-score computed using 1,000 bootstrap samples.

Class	Pr (95% CI)	Recall (95% CI)	F1-score (95% CI)
BH	0.850–1.000	0.852–1.000	0.884–1.000
BU	0.800–1.000	0.903–1.000	0.868–0.989
CH	0.925–0.992	0.961–1.000	0.950–0.991
CPM	0.945–0.994	0.958–1.000	0.960–0.992
TSL	0.969–0.996	0.969–0.997	0.974–0.994
TSM	0.955–0.993	0.967–0.996	0.967–0.991
TTS	1.000–1.000	0.962–0.995	0.980–0.998
TTM	0.820–0.967	0.937–1.000	0.889–0.977
TTY	0.990–0.999	0.975–0.992	0.984–0.994

To ensure the statistical reliability and robustness of the model’s performance metrics, we computed 95% confidence intervals (CIs) for each disease class using non-parametric bootstrapping with 1,000 resamples on the test predictions. These intervals quantify the expected variability of precision, recall, and F1-score due to sampling variation, providing a more comprehensive understanding of model performance beyond point estimates alone.

Unlike aggregate accuracy, which can mask class-level discrepancies, the class-wise CIs reveal the predictive stability across individual diseases, especially for visually similar leaf symptoms. For instance, classes with fewer test samples exhibit slightly wider confidence intervals, reflecting higher variability, whereas classes with larger sample sizes demonstrate tighter bounds. This observation supports the conclusion that the model performs consistently well across all disease classes, while also acknowledging natural variation. The confidence intervals, presented in Table 12, confirm that the high classification scores (99%+) are statistically significant and not due to overfitting or data leakage. Incorporating these statistical bounds enhances the transparency and credibility of the results, addressing concerns related to model overconfidence in high-stakes agricultural disease detection. The separation of confusion matrices by plant type (Banana, Cherry, and Tomato) further illustrates that all models achieve high classification accuracy, with the proposed model consistently showing superior performance and the fewest misclassifications.

### Grad-CAM visualization

**Fig. 11:**
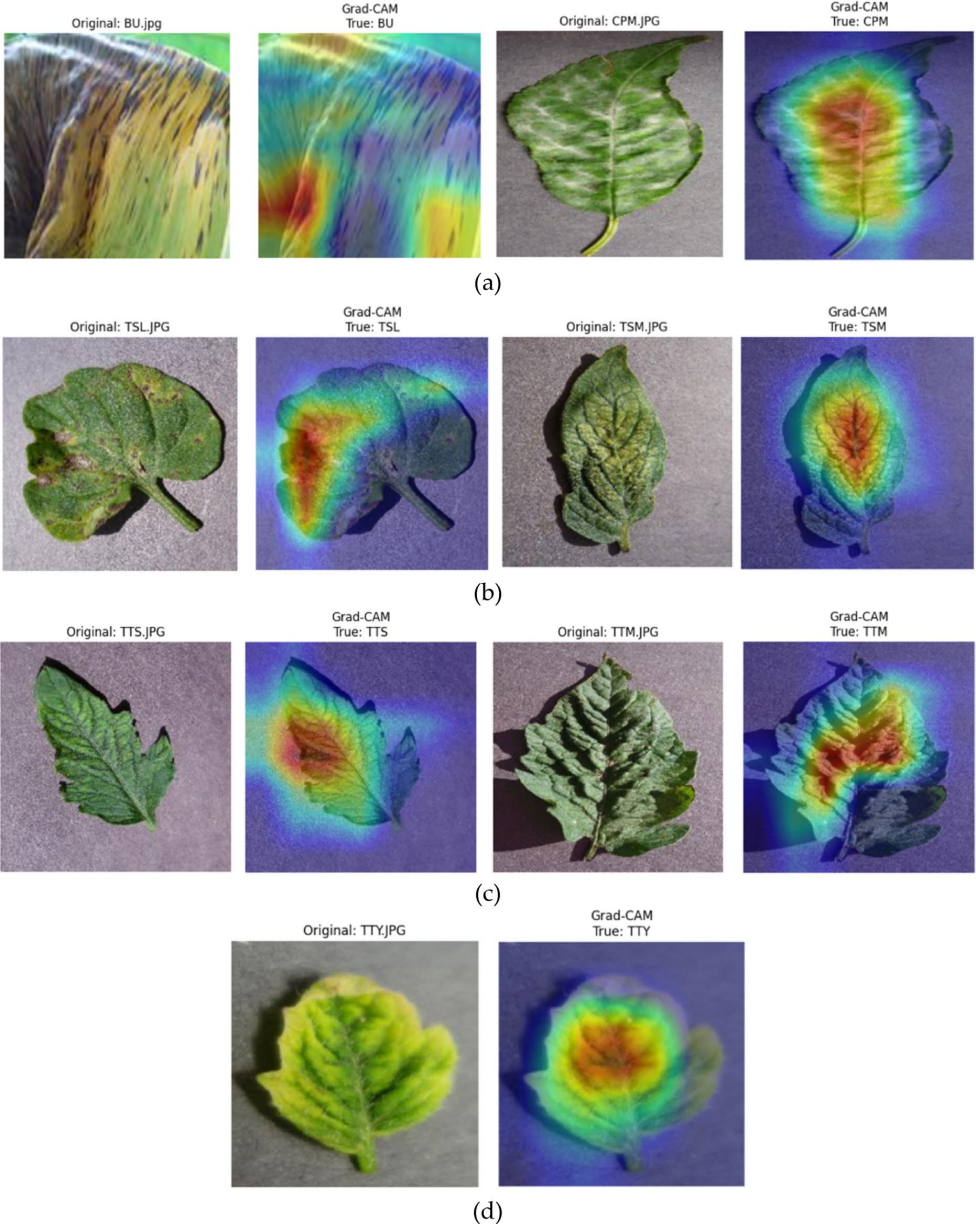
Grad-CAM visualization of 3-plant leaf diseases (**a**) BU and CPM, (**b**) TSL and TSM, (**c**) TTS and TTM, (**d**) TTY.

The visualizations shown in Fig. 11 illustrate using Grad-CAM (Gradient-weighted Class Activation Mapping) to interpret deep learning model predictions for plant leaf disease detection, specifically for banana, cherry, and tomato leaves. Each subfigure (a–d) pairs the original input leaf image with its corresponding Grad-CAM heatmap. These heatmaps highlight the most influential regions of the leaf that contributed to the model’s classification decision. The heatmaps are colour-coded maps where the red areas correspond to regions with the highest impact on our prediction, and the blue areas are regions with a minor impact. For example, in the above illustration, for BU, the Grad-CAM highlights those streaked and darkened areas of the banana leaf. Likewise, for diseases such as TSL and TTM, the heatmaps concentrate on spots or texture anomalies typical of these diseases. This interpretability is key to trustworthy AI in agriculture since it validates the model’s focus and helps the plant pathologist and the farmer know what symptoms the AI model is looking for. By aligning visual cues with expert knowledge, Grad-CAM facilitates the deployment of deep learning-based models in plant disease warning systems. Strictly speaking, GCAM computes the gradient of the target label concerning the activation map of a convolutional layer and multiplies the activation map by the average gradient in each channel across the spatial dimensions. The outcome is a class-discriminative localization map that reveals the model’s most discriminative image regions for making the classification. The blue-to-red color-coded heatmaps show which parts of the leaf were the most influential for the classification. For instance, in the case of the BU class, the model focuses on streaks and lesions. In tomato classes such as TSL or TTM, Grad-CAM correctly highlights symptomatic regions like necrotic spots or textured patches. This interpretability technique is crucial for model transparency and trustworthiness, especially in sensitive domains like agricultural diagnostics, where incorrect classifications can lead to poor crop management decisions. Grad-CAM thus serves as a visual explanation tool and a validation method for model reliability, potentially facilitating human-in-the-loop systems where plant pathologists can cross-verify model outputs.

## Ablation study

The ablation study presented in the document highlights the architectural, computational, and performance-driven justifications for adopting the proposed Hybrid ConvNet-ViT model over traditional pre-trained models such as EfficientNetV2, ConvNeXt, Swin Transformer, and ViT. The study provides a systematic breakdown of model configurations including input size, FLOPs, parameter count, learning rate, and architectural type illustrating how these design choices affect model complexity and efficiency. From a computational perspective, the proposed hybrid model maintains a moderate computational footprint with 9–12 GFLOPs and 32–35 million parameters, which strikes a balance between lightweight ConvNets like ConvNeXt (~ 4.5 GFLOPs) and high-capacity Transformers like ViT (~ 17.5 GFLOPs, ~ 86 M parameters). Despite having fewer parameters than ViT, the proposed model surpasses its accuracy, achieving 99.29% compared to ViT’s 98.92%, demonstrating that the hybrid approach is more parameter-efficient. Regarding learning dynamics, the proposed model benefits from a carefully chosen learning rate (0.0005), which stabilizes training when combining ConvNet-based and transformer-based layers. Ablation comparisons reveal that pure ConvNet (EfficientNetV2, ConvNeXt) tend to underperform on global contextual relationships due to their local receptive fields, while pure transformers (ViT, Swin) struggle with fine-grained local feature extraction and require more data and computation. The hybrid model mitigates these weaknesses by utilizing ConvNet layers to capture local patterns (like disease spots or edges) and ViT layers to model broader spatial dependencies across the entire leaf surface. Importantly, the ablation study confirms that no single component ConvNet or Transformer can individually deliver the same level of performance as the integrated hybrid model. The synergy between these two architectural paradigms enables superior generalization across varied crop types (banana, cherry, tomato) and disease conditions, even under noisy, complex backgrounds. Overall, the ablation study justifies the proposed model’s architecture as a novel fusion of ConvNet and ViT and a practically deployable solution with optimal trade-offs between computational cost and classification performance. This positions the proposed model as a scalable, accurate, and efficient alternative to standalone pre-trained models for real-world agricultural disease detection.

**Table 13:** Ablation study on model variants, ViT exclusion, layer freezing, and learning rate effects.

Configuration	Acc (%)	F1-score (%)	Params (M)	GFLOPs
EfficientNetV2 only	97.88	97.6	24	8.4
ConvNeXt only	97.65	97.32	28	4.5
Swin transformer only	97.21	96.9	29	4.5
ViT only	97.92	97.71	86	17.5
EffNet + ConvNeXt	98.18	97.89	52	12.9
EffNet + Swin	98.04	97.8	53	13.2
EffNet + ViT	98.39	98.05	59	13.6
ConvNeXt + Swin	97.98	97.63	57	13.4
ConvNeXt + ViT	98.41	98.07	64	14
Swin + ViT	98.28	97.91	68	14.4
EffNet + ConvNeXt + ViT	98.72	98.33	76	15.3
EffNet + Swin + ViT	98.65	98.25	80	15.6
ConvNeXt + Swin + ViT	98.58	98.19	83	15.8
All ConvNet branches only (no ViT)	98.02	97.85	35	9.1
All ConvNet + ViT (full hybrid)	99.29	99.18	33	12
Full hybrid - ViT head removed	98.51	98.36	33	11.5
Full hybrid − 50% layers frozen	98.88	98.85	33	12
Full hybrid - LR = 0.001	98.76	98.7	33	12
Full hybrid - LR = 0.0001	98.93	98.9	33	12

The detailed ablation results are shown in Table 13, which evaluates different architectural configurations and training setups. The table compares individual backbones, 2- and 3-branch combinations, and the impact of removing the ViT head. It also examines sensitivity to frozen-layer ratios and learning rates. As indicated, the full hybrid model achieved the highest performance (99.29% accuracy, 99.18% F1-score), while variants with components removed or changed showed small to moderate decreases, confirming the significance of each design choice. To evaluate the contribution of each architectural element within the proposed hybrid model, we conducted an extended ablation study as shown in Table 13. This analysis isolates the performance impact of individual backbones (EfficientNetV2, ConvNeXt, Swin Transformer, and ViT), evaluates partial combinations (two- and three-branch subsets), and examines the effect of removing the ViT head. Additionally, we assess the model’s sensitivity to key training settings, including frozen-layer ratios and learning rate variations. The results demonstrate that while each component contributes positively, the full hybrid model consistently outperforms all configurations, confirming the synergistic benefit of combining local and global representations. Moreover, sensitivity tests indicate the model maintains stable performance across reasonable training variations, reinforcing its robustness.

While it is acknowledged that the proposed Hybrid ConvNet-ViT model contains approximately 32–35 million parameters and operates up to 12 GFLOPs, significantly higher than lighter backbones like ConvNeXt-T (~ 4.5 M, ~ 4.5 GFLOPs) or EfficientNet-B3 (~ 10 M, ~ 1.8 GFLOPs) the design choice is driven by more than just raw accuracy improvement. Despite a modest increase of 0.37–0.44% points in testing accuracy (Table 11), the proposed model exhibits superior generalization across diverse disease types and improved robustness under real-world imaging conditions, as evidenced by consistently higher metrics across 5-fold cross-validation (Table 10) and all five-evaluation metrics during testing. Moreover, Grad-CAM visualizations (Fig. 11) validate the model’s ability to focus more precisely on disease-affected regions, which lightweight models often struggle to capture due to their limited capacity. The hybrid architecture’s combination of ConvNet-local features and ViT-global representations ensures a balanced trade-off between interpretability, robustness, and accuracy, critical for high-stakes agricultural diagnostics. Therefore, the ~ 3× increase in model size is justified by the improved model reliability, decision transparency, and cross-crop scalability, rather than accuracy alone.

## Discussion

In this work, we introduced a novel Hybrid ConvNet-ViT model designed to address the challenges in tomato leaf disease classification by leveraging the complementary strengths of ConvNet and ViTs. This hybrid architecture combines the powerful local feature extraction capabilities of ConvNets with the global context modelling of transformers, resulting in a more comprehensive understanding of complex image features such as subtle disease symptoms and variations across leaf textures. To evaluate the effectiveness of our proposed model, we conducted a comparative analysis against several state-of-the-art (SOTA) models, as summarized in Table 14.

**Table 14:** Classification accuracy comparison of proposed and SOTA models.

Authors	Model	Acc (%)
^17^	EfficientNetB0	87.83
^19^	Hybrid ConvNet	91.17
^36^	VGG19	99.16
^37^	ConvNet	95.81
^38^	MobileNet ConvNet	98.60
^39^	FCDCONVNET	98.02
^40^	ToLeD	91.21
^41^	RConvNet	96.73
^42^	SNDPN	97.59
Proposed	Hybrid ConvNet-ViT	99.29

**Fig. 12:**
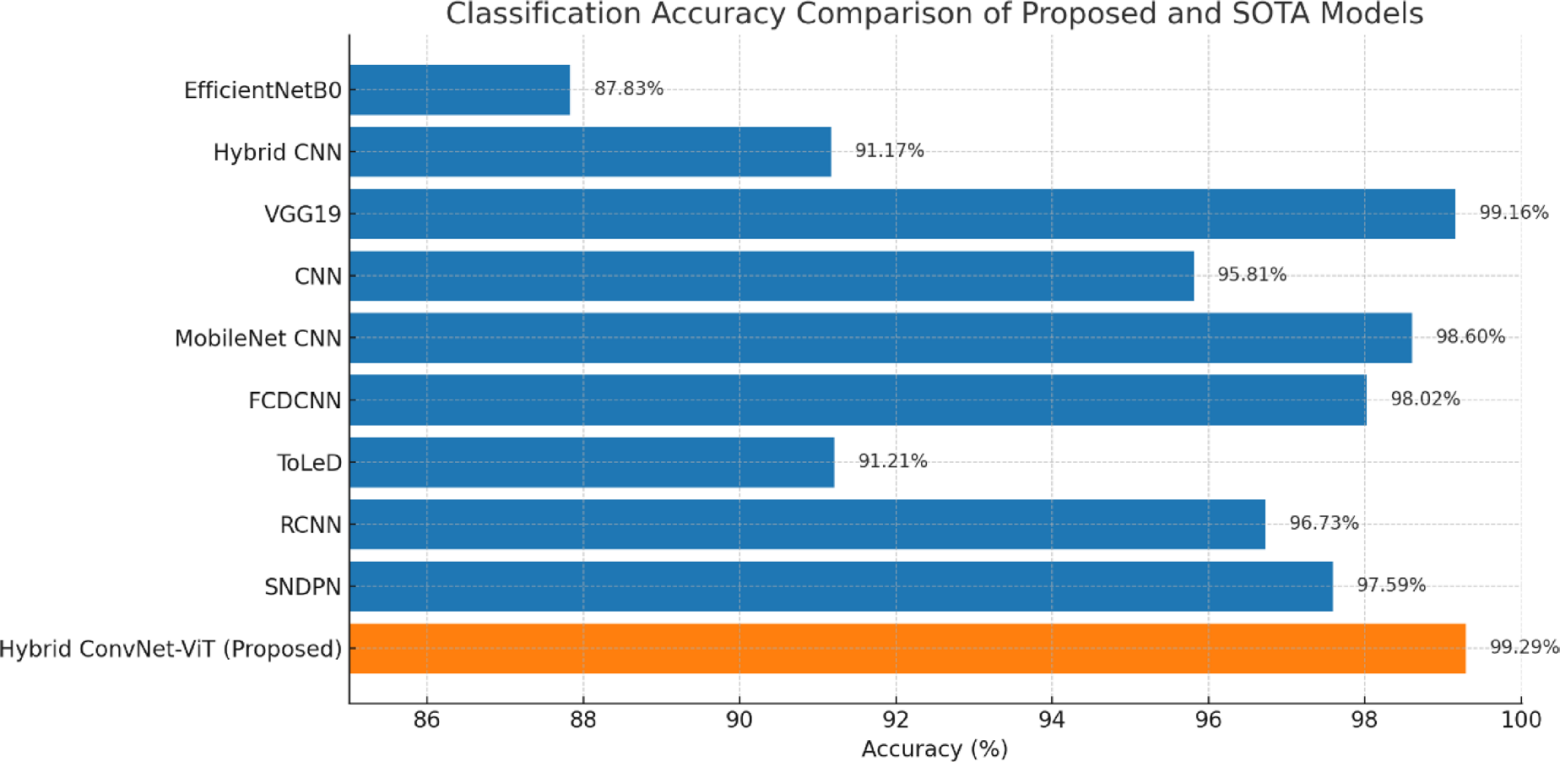
Accuracy comparison of proposed and other SOTA models.

The results demonstrate the superior performance of the Hybrid ConvNet-ViT, which achieved a classification accuracy of 99.29%, the highest among all evaluated models. For comparison, VGG19 a well-established deep ConvNet reached 99.16% accuracy, while MobileNet ConvNet and FCDConvNet achieved 98.60% and 98.02%, respectively. Other conventional architectures, such as EfficientNetB0 (87.83%), Hybrid ConvNet (91.17%), and ToLeD (91.21%), showed notably lower performance. More advanced models like RCONVNET and SNDPN reported accuracies of 96.73% and 97.59%, respectively, yet still fell short of the performance achieved by our hybrid model. These findings highlight the advantages of integrating convolutional and transformer-based techniques in disease classification tasks. The Hybrid ConvNet-ViT model improves classification precision and enhances robustness in handling diverse and challenging real-world agricultural conditions, such as varying lighting, backgrounds, and disease severity levels. The high accuracy achieved by our model suggests its strong potential for practical deployment in intelligent agricultural systems, where early and accurate disease detection is crucial for improving crop management, reducing losses, and supporting sustainable farming practices. Figure 12 depicts the proposed and other SOTA models classification accuracy comparison.

## Conclusion

This study introduced a novel Hybrid ConvNet-ViT architecture for robust and accurate classification of crop leaf diseases in banana, cherry, and tomato plants. By strategically integrating convolutional layers for local feature extraction with vision transformer layers for global context understanding, the proposed model addresses the limitations of standalone ConvNet and transformer-based models. Comprehensive experiments, including 5-fold cross-validation and testing phases, demonstrated the superiority of the hybrid model over state-of-the-art pre-trained architectures such as EfficientNetV2, ConvNeXt, Swin Transformer, and ViT. The hybrid model achieved the highest testing accuracy of 99.29%, along with outstanding sensitivity, specificity, precision, and F1 score. Grad-CAM visualizations further validated the model’s interpretability, highlighting the critical leaf regions contributing to disease classification. The ablation study confirmed that the hybrid architecture delivers an optimal balance between computational efficiency and predictive accuracy. These findings underscore the potential of the Hybrid ConvNet-ViT model as a practical and scalable solution for real-world agricultural applications, empowering early disease detection, informed decision-making, and sustainable crop management.

However, the study also has a few limitations. The model relies entirely on image-based inputs, which may be affected by variations in lighting, background clutter, or image quality common challenges in real-world field conditions. Additionally, while the dataset includes three major crop types, its applicability to a broader range of plants or less common diseases remains untested. The current model also requires a moderate computational setup, which could be a barrier for direct deployment on low-resource devices without further optimization. Moreover, the approach focuses solely on classification and does not currently provide disease severity estimation or actionable treatment suggestions. Furthermore, all current experiments are based on an internally split dataset derived from a single distribution. Although 5-fold cross-validation ensures internal robustness, the model’s generalizability to unseen datasets remains an open question. Future work will focus on evaluating the Hybrid ConvNet-ViT architecture on cross-dataset setups, such as testing on PlantDoc or additional disease categories from PlantVillage, to rigorously assess external validity across diverse image domains and conditions.

While the proposed model demonstrates strong performance, several directions can be explored to enhance its utility and adaptability further. First, integrating real-time disease detection capabilities via deployment on mobile devices or drones could provide on-field diagnostic support for farmers. Second, expanding the dataset to include additional plant species and disease types would improve the model’s generalizability and robustness. Additionally, incorporating multi-modal data, such as thermal or hyperspectral imagery, may enhance classification under complex environmental conditions. Future work can also explore lightweight versions of the hybrid architecture for edge computing applications and investigate self-supervised or few-shot learning techniques to reduce the dependency on large, labeled datasets. Finally, combining disease detection with severity estimation and treatment recommendations could lead to developing an end-to-end precision agriculture platform.

## Data Availability

The datasets used during the current study are available from the corresponding author on reasonable request.
